# Hemodynamic forces prevent myxomatous valve disease in mice through KLF2/4 signaling

**DOI:** 10.1172/JCI186593

**Published:** 2025-06-16

**Authors:** Jesse A. Pace, Lauren M. Goddard, Courtney C. Hong, Liqing Wang, Jisheng Yang, Mei Chen, Yitian Xu, Martin H. Dominguez, Siqi Gao, Xiaowen Chen, Patricia Mericko-Ishizuka, Can Tan, Tsutomu Kume, Wenbao Yu, Kai Tan, Wayne W. Hancock, Giovanni Ferrari, Mark L. Kahn

**Affiliations:** 1 Cardiovascular Institute, Department of Medicine, Perelman School of Medicine, University of Pennsylvania, Philadelphia, Pennsylvania, USA.; 2 Division of Transplant Immunology, Department of Pathology and Laboratory Medicine, and Biesecker Center for Pediatric Liver Disease, Children’s Hospital of Philadelphia and University of Pennsylvania, Philadelphia, Pennsylvania, USA.; 3 Department of Medicine, Feinberg Cardiovascular and Renal Research Institute, Feinberg School of Medicine, Northwestern University, Chicago, Illinois, USA.; 4 Division of Oncology and Center for Childhood Cancer Research, Center for Single Cell Biology, Children’s Hospital of Philadelphia, Philadelphia, Pennsylvania, USA.; 5 Department of Pediatrics, Perelman School of Medicine, University of Pennsylvania, Philadelphia, Pennsylvania, USA.; 6 Department of Surgery, Department of Biomedical Engineering, Columbia University, New York, New York, USA.

**Keywords:** Cardiology, Vascular biology, Cardiovascular disease, Endothelial cells

## Abstract

Myxomatous valve disease (MVD) is the most common form of cardiac valve disease in the developed world. A small fraction of MVD is syndromic and arises in association with matrix protein defects such as those in Marfan syndrome, but most MVD is acquired later in life through an undefined pathogenesis. The KLF2/4 transcription factors mediate endothelial fluid shear responses, including those required to create cardiac valves during embryonic development. Here we test the role of hemodynamic shear forces and downstream endothelial KLF2/4 in mature cardiac valves. We find that loss of hemodynamic forces in heterotopically transplanted hearts or genetic deletion of KLF2/4 in cardiac valve endothelium confers valve cell proliferation and matrix deposition associated with valve thickening, findings also observed in mice expressing the mutant fibrillin-1 protein known to cause human MVD. Transcriptomic and histologic analysis reveals increased monocyte recruitment and TGF-β signaling in both fibrillin-1–mutant valves and valves lacking hemodynamic forces or endothelial KLF2/4 function, but only loss of TGF-β/SMAD signaling rescued myxomatous changes. We observed reduced KLF2/4 expression and augmented SMAD signaling in human MVD. These studies identify hemodynamic activation of endothelial KLF2/4 as an environmental homeostatic regulator of cardiac valves and suggest that non-syndromic MVD may arise in association with disturbed blood flow across the aging valve.

## Abstract 2

Blood flow across the heart valve maintains normal valve structure and function through signals in the valve cells that contact blood.

## Graphical abstract

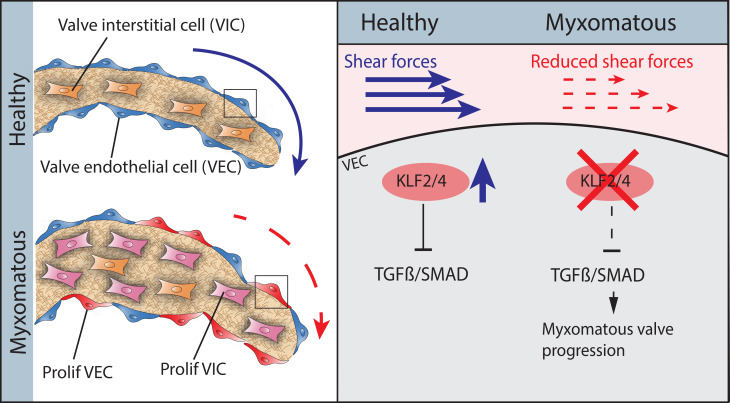

## Introduction

The cardiac valves open and close over 100,000 times a day to ensure unidirectional, forward blood flow through the pumping heart. Primary valvular heart disease affects approximately 2.5% of individuals when assessed by echocardiography, and is a common cause of heart failure (1, 2). Valvular heart disease often requires valve repair or replacement via open heart surgery or percutaneous approaches (1). Myxomatous valve disease (MVD), typically affecting the mitral valve, is the most common type of acquired valvular heart disease (2). MVD is characterized by leaflet thickening and degeneration, ultimately leading to regurgitant blood flow and valve prolapse (3). The mature valve leaflet has a trilaminar structure in which each layer has a distinct matrix and cellular composition and biomechanical features. The myxomatous valve exhibits increased deposition of matrix proteins such as glycosaminoglycans and increased cellular content, changes that ultimately impair its mechanical function.

Insight into the pathogenesis of MVD has primarily come from examination of a small number of inherited, genetic syndromes that affect extracellular matrix proteins. The most prominent of these is Marfan syndrome that arises in association with mutations in the gene encoding fibrillin-1 (FBN1), a secreted extracellular matrix protein. Marfan syndrome is associated with both MVD and aortic aneurysm due to matrix changes in the cardiac valve, the aortic root, and the ascending aorta (4–6). Mouse models of Marfan syndrome have demonstrated increased TGF-β/Smad signaling in myxomatous valve pathophysiology, consistent with a role for FBN1 in limiting TGF-β activity in the extracellular matrix (7–9). Analysis of human and canine valve specimens has also demonstrated increased activation of TGF-β/Smad signaling in both syndromic and non-syndromic myxomatous valves and mitral valve prolapse, suggesting that TGF-β/Smad signaling may play a causal role in non-syndromic MVD (10–14). However, since the vast majority of MVD is acquired later in life and not associated with a primary matrix protein defect, whether and how the syndromic and non-syndromic MVD might reflect a shared pathogenic mechanism remains unknown.

Cardiovascular development and function are tightly associated with the hemodynamic forces generated by the beating heart, especially fluid shear forces generated at the point of contact between flowing blood and the cardiovascular structures that contain it. Studies performed over the past two decades have established that the related Krüppel-like transcription factors KLF2 and KLF4 are expressed by endothelial cells in response to hemodynamic shear, and mediate many of the known effects of such forces on endothelial cells (15–19). We have previously shown that, in the developing heart, KLF2/4 expression by endothelial cells that overlie the cardiac cushions orchestrates the remodeling of cushions into valves in direct response to hemodynamic shear forces (19). In mature vessels, endothelial KLF2/4 expression is active at sites of fluid shear and required to maintain an antiinflammatory vascular phenotype and vascular integrity (20, 21). Whether hemodynamic forces sustain KLF2/4 expression in the mature cardiac valve and play an ongoing homeostatic role in the valve is not known.

In the current study we use a mouse heterotopic heart transplant model to demonstrate that hemodynamic forces are required to maintain valvular endothelial KLF2/4 expression and prevent myxomatous valve changes. Genetic deletion of KLF2/4 specifically in cardiac valve endothelial cells conferred rapid myxomatous changes, demonstrating a causal link between hemodynamic forces, valve endothelial cell KLF2/4 expression, and prevention of MVD. Mechanistically, loss of valve endothelial KLF2/4 was associated with rapid activation of TGF-β signaling and recruitment of CCR2^+^ monocytes. Genetic loss of TGF-β signaling significantly slowed myxomatous changes, but blockade of monocyte recruitment failed to prevent myxomatous changes conferred either by loss of KLF2/4 or by expression of the mutant FBN1 C1039G protein associated with Marfan syndrome. Finally, human myxomatous mitral valve specimens exhibited decreased endothelial KLF2/4 expression and increased TGF-β signaling compared with non-myxomatous controls. These studies identify a critical homeostatic role for hemodynamic forces and endothelial KLF2/4 expression in the mature heart valve and suggest that acquired MVD may arise due to disruption of this pathway in the aging heart.

## Results

### Hemodynamic forces control KLF2/4 expression in the mouse mitral valve.

**Figure 1:**
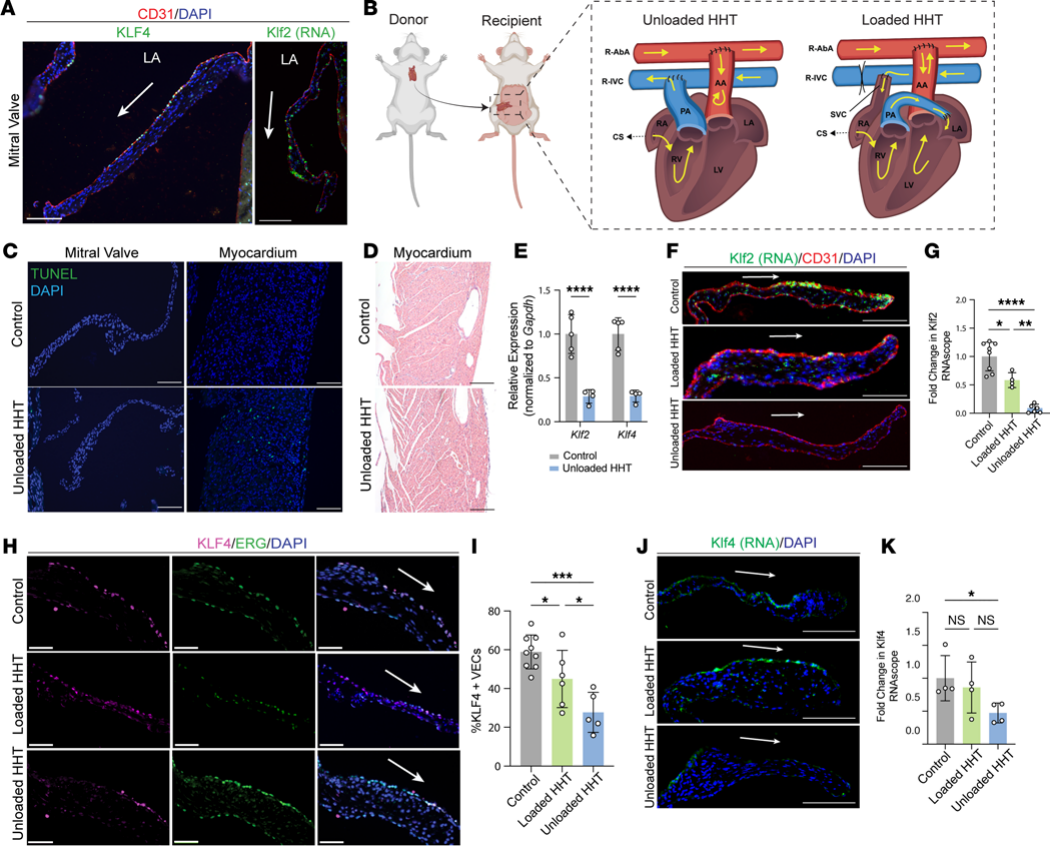
Hemodynamic forces control KLF2/4 expression in adult valve endothelial cells. (**A**) Immunostaining for KLF4 (left) or in situ hybridization for *Klf2* mRNA (right) costained with the endothelial marker CD31 and DAPI in mitral valve of adult mouse. Scale bars: 50 μm. (**B**) Schematic of unloaded and loaded heterotopic heart transplant (HHT) model in which the recipient abdominal aorta (R-AbA) is anastomosed to the donor ascending aorta (AA). The pulmonary artery (PA) of the donor heart is anastomosed to the recipient inferior vena cava (R-IVC) to allow for venous drainage of the donor heart. CS, coronary sinus; LA, left atrium; LV, left ventricle; RA, right atrium; RV, right ventricle. (**C**) TUNEL staining in mitral valve and myocardium of control and donor hearts 4 days after transplant. Scale bars: 50 μm. (**D**) H&E staining of myocardium at 4 days after HHT. Scale bars: 50 μm. (**E**) Quantitative PCR measurement of *Klf2* and *Klf4* mRNA expression using isolated mitral valve tissue from control and unloaded HHT hearts. ****P < 0.0001, by unpaired *t* tests. (**F**) In situ hybridization for *Klf2* mRNA costained with CD31 and DAPI in mitral valves of control, loaded HHT, and unloaded HHT hearts 4 days after transplant was performed using RNAscope. Scale bars: 50 μm. (**G**) Quantification of *Klf2* percentage area stained in **F**. *P < 0.05, **P < 0.01, ****P < 0.0001, by 2-way ANOVA with Tukey’s multiple-comparison tests. (**H**) Immunostaining for KLF4, ERG, and DAPI in mitral valves of control, loaded HHT, and unloaded HHT hearts 4 days after transplant. Scale bars: 50 μm. (**I**) Quantification of immunostaining in **H**. The percentage of valve endothelial cells (VECs), based on ERG expression, that are KLF4^+^ is shown. *P < 0.05, ***P < 0.001, by 2-way ANOVA with Tukey’s multiple-comparison tests. (**J**) In situ hybridization for *Klf4* mRNA costained with DAPI in mitral valves of control, loaded HHT, and unloaded HHT hearts 4 days after transplant was performed using RNAscope. Scale bars: 50 μm. (**K**) Quantification of *Klf4* percentage area stained in **J**. *P < 0.05, by 2-way ANOVA with Tukey’s multiple-comparison tests.

Endothelial expression of KLF2 and KLF4 is activated by hemodynamic shear forces in zebrafish, mice, humans, and cultured cells, and we have recently demonstrated that shear-regulated KLF2 controls cardiac valve development in the mouse (15–19). To determine whether KLF2/4 transcription factor expression remains active in the mature cardiac mitral valve, we immunostained for KLF4. KLF4 colocalized with the endothelial cell marker PECAM-1 along both sides of the cardiac valve, although it appeared stronger along the side of the valve that faces forward blood flow (“flow side”) (Figure 1A). In situ hybridization for *Klf2* mRNA revealed a similar expression pattern, with enrichment of *Klf2* on the flow side of the valve (Figure 1A). These findings reveal that KLF2/4 expression is maintained in mature cardiac valve endothelial cells (VECs) in a pattern consistent with regulation by hemodynamic shear forces in the beating heart.

To test whether VEC KLF4 expression is driven by hemodynamic forces in the mature heart, we used a previously reported heterotopic heart transplant (HHT) model (22–24). In the HHT model a donor heart is transplanted into the abdomen of a syngeneic recipient such that the donor ascending aorta is anastomosed to the recipient abdominal aorta and the donor pulmonary artery to the recipient inferior vena cava (Figure 1B). In this arrangement, retrograde flow into the donor ascending aorta maintains coronary artery perfusion and oxygenation of the donor heart. The left atrium is tied off, eliminating blood flow across the mitral valve, while blood flow across the tricuspid valve is reduced to coronary sinus venous outflow (Figure 1B). Thus, in the fully unloaded HHT model there is no flow across the mitral valve and significantly reduced flow across the tricuspid valve. Analysis of donor hearts 4 days after HHT using terminal deoxynucleotidyl transferase–mediated dUTP nick end labeling (TUNEL) immunostaining demonstrated that valve cells were not undergoing apoptosis but identified scattered apoptotic cells within the myocardium and no obvious histologic changes (Figure 1, C and D). To assess hypoxia, we performed immunostaining for HIF1α in HHT hearts and native heart controls 1 month after transplant and found no evidence of increased HIF1α staining (Supplemental Figure 1A; supplemental material available online with this article; https://doi.org/10.1172/JCI186593DS1). Additionally, we performed quantitative reverse transcriptase PCR on isolated mitral valve, which showed no significant changes in *Hif1a* and *Vegfa* (Supplemental Figure 1, B and C). Following HHT, the donor heart volume shrank dramatically as a result of loss of preload required to fill the heart, but histologic studies failed to demonstrate ischemic changes in the myocardium (Figure 1D). To examine changes in KLF2/4 in the unloaded setting, we dissected mitral valve tissue 4 days after unloading with HHT and performed quantitative reverse transcriptase PCR for *Klf2 and Klf4* (Figure 1E). We detected an approximately 70% decrease in both *Klf2* and *Klf4* in comparison with control mitral valve (Figure 1E). Consistent with these quantitative PCR studies, in situ hybridization for *Klf2* mRNA revealed a greater than 80% decrease in *Klf2* expression 4 days after unloading with HHT (Figure 1, F and G). Additionally, immunostaining for KLF4 and ERG, a nuclear endothelial cell marker, revealed a greater than 50% reduction in KLF4^+^ VECs 4 days after HHT (Figure 1, H and I). To test whether KLF2/4 expression tightly reflects hemodynamic shear forces across the valve endothelium, and more rigorously control for effects of the transplant procedure, we used a modified HHT model in which the donor pulmonary artery is anastomosed to the left atrium to allow for partial loading of the donor heart with restored blood flow across the mitral valve (“loaded HHT”; Figure 1B). Immunostaining for KLF4 and in situ hybridization for *Klf2 and Klf4* mRNA revealed that both endothelial KLF4 and *Klf2/4* expression in the loaded HHT mitral valve was decreased to a level that was intermediate between expression in the control and that in the fully unloaded HHT heart (Figure 1, F–K). These studies support tight regulation of the KLF2 and KLF4 transcription factors by hemodynamic flow across the mature heart valve.

### Hemodynamic unloading drives myxomatous valve formation.

**Figure 2:**
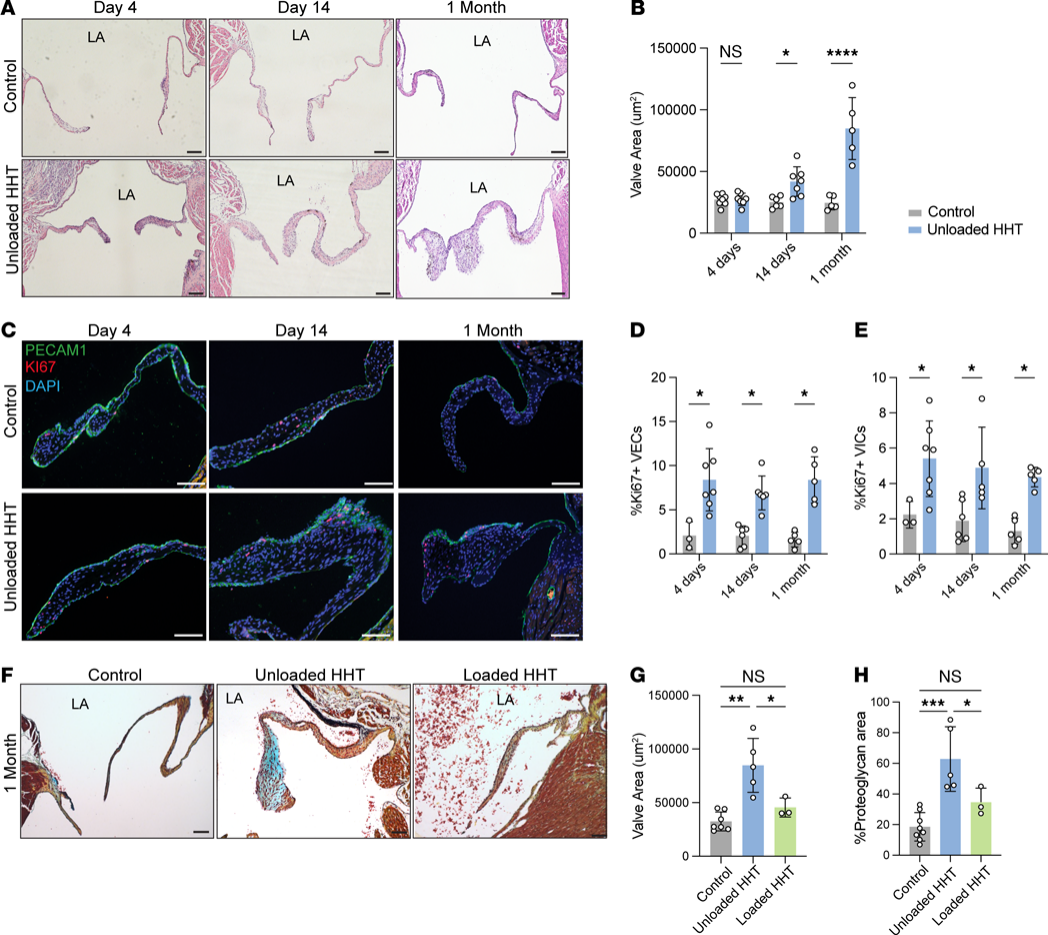
Hemodynamic unloading leads to formation of myxomatous valves. (**A**) H&E staining of mitral valve at 4 days, 14 days, and 1 month after HHT. Scale bars: 50 μm. (**B**) Quantification of mitral valve leaflet area after HHT. Each point represents a single mouse. *P < 0.05, ****P < 0.0001, by unpaired *t* tests. (**C**) Immunostaining for Ki67 and PECAM-1 in mitral valves of control and unloaded HHT hearts 4 days after transplant. Scale bars: 50 μm. (**D**) Percentage of Ki67^+^PECAM-1^+^ cells in mitral valves of control and unloaded HHT hearts. *P < 0.05, by unpaired *t* tests. (**E**) Percentage of Ki67^+^PECAM-1^–^ cells in mitral valves of control and unloaded HHT hearts. *P < 0.05, by unpaired *t* tests. (**F**) Movat’s pentachrome staining of mitral valves 1 month after HHT. Scale bars: 50 μm. (**G**) Quantification of valve leaflet area after loaded and unloaded HHT. *P < 0.05, **P < 0.01, by 2-way ANOVA with Tukey’s multiple-comparison tests. (**H**) Quantification of valve proteoglycan area based on Alcian blue area in **F**. *P < 0.05, ***P < 0.001, by 2-way ANOVA with Tukey’s multiple-comparison tests.

Endothelial KLF2/4 expression is considered a primary mechanism by which hemodynamic forces maintain homeostasis in the vasculature and prevent pathogenic states such as atherosclerosis (20, 21, 25, 26), but whether they serve a similar role in cardiac valves is unknown. We therefore further investigated the effect of decreased hemodynamic forces and reduced endothelial KLF2/4 expression in the mitral valve using the HHT model. Hearts were harvested after 4 days, 14 days, or 1 month of hemodynamic unloading, and hematoxylin and eosin (H&E) staining was performed to characterize the valve leaflets (Figure 2A). H&E staining revealed progressive thickening of the mitral valve leaflets compared with those of the control recipient heart, with quantified leaflet area significantly increased starting at 14 days after unloading and loss of hemodynamic forces (Figure 2B). Immunostaining for the proliferative marker Ki67 and PECAM-1, an endothelial cell marker, revealed increased proliferation of both PECAM-1^+^ VECs and PECAM-1^–^ valve interstitial cells (VICs) starting at 4 days after transplant (Figure 2, C–E). These histologic changes were similar to those associated with myxomatous degeneration, which is characterized by increased leaflet size, increased VEC and VIC proliferation, and accumulation of glycosaminoglycan (GAG) matrix that is detectable using Movat’s pentachrome staining (3). Indeed, comparison of Movat’s staining in unloaded hearts and control hearts 1 month after transplant revealed increased GAG matrix as well as valve thickening and proliferation (Figure 2, F and G). Consistent with the changes in KLF2/4 expression, intermediate changes in valve thickening and valve area were noted following loaded HHT (Figure 2, F and G). Quantitation of valve proteoglycan content based on Alcian blue staining revealed that mitral valve proteoglycan area was significantly increased in the unloaded HHT but not in the loaded HHT (Figure 2H). The partially loaded HHT model controls for the cardiac transplant procedure and strengthens the conclusion that hemodynamic conditions are the driver of valvular changes. These studies reveal that loss of hemodynamic forces is sufficient to confer a human MVD phenotype in association with reduced VEC expression of KLF2 and KLF4.

Altered valve endothelial cell junctions have been recently reported to play an important role in maintenance of mitral valve integrity (27). To assess for potential changes in VEC junctions, we performed whole-mount immunostaining for cell junction markers CD31 and CDH5 in mitral valves 4 days after transplant (Supplemental Figure 2, A and B). Quantification of reticular adherens junctions in unloaded HHT mitral valves and control mitral valves revealed a slight increase in the percentage of endothelial cells containing reticular adherens junctions, although this difference was not statistically significant (*P* = 0.076322, *P* = 0.079923; Supplemental Figure 2C). Thus it is unlikely that changes in VEC junctions primarily underlie the myxomatous phenotype observed in the hemodynamically unloaded valve.

### Genetic loss of KLF2/4 in adult heart valve endothelium results in myxomatous valve formation.

**Figure 3:**
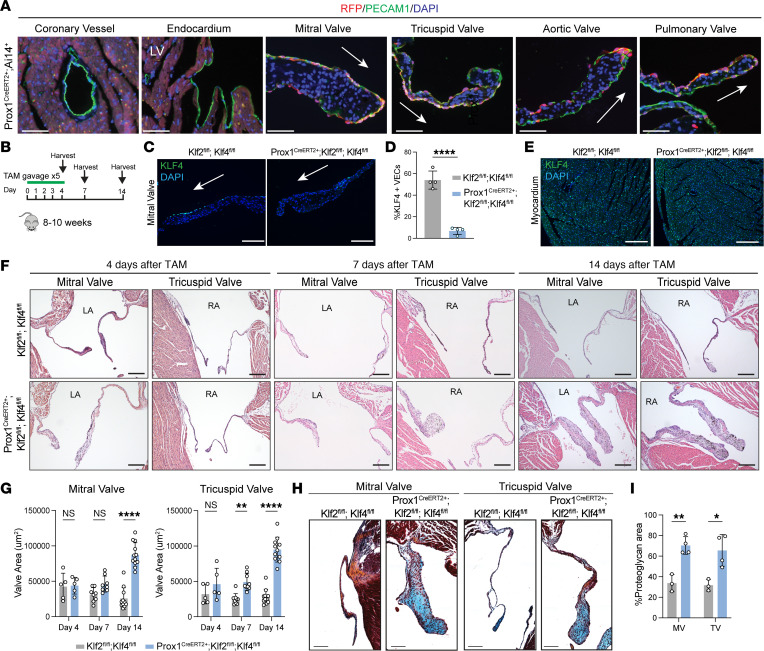
Genetic loss of endothelial KLF2/4 in the mature heart valve results in myxomatous valve formation. (**A**) Immunostaining for RFP, PECAM-1, and DAPI in *Prox1^CreERT2+^* *Ai14* mice after 5 days of tamoxifen treatment. Scale bars: 100 μm. (**B**) Schematic of tamoxifen treatment and tissue collection in *Prox1^CreERT2+^* *Klf2^fl/fl^* *Klf4^fl/fl^* mice. (**C**) Immunostaining for KLF4 in mitral valve from *Prox1^CreERT2+^* *Klf2^fl/fl^* *Klf4^fl/fl^* and *Klf2^fl/fl^* *Klf4^fl/fl^* control animals at 4 days after tamoxifen treatment. (**D**) Quantification of KLF4 immunostaining in mutant and control mitral valves. *N* = 5 mice per group. ****P < 0.0001. (**E**) Immunostaining for KLF4 in the myocardium of hearts from *Prox1^CreERT2+^* *Klf2^fl/fl^* *Klf4^fl/fl^* and *Klf2^fl/fl^* *Klf4^fl/fl^* animals at 4 days after tamoxifen treatment. (**F**) H&E staining of heart tissue from *Prox1^CreERT2+^* *Klf2^fl/fl^* *Klf4^fl/fl^* and *Klf2^fl/fl^* *Klf4^fl/fl^* mice at days 4, 7, and 14 after tamoxifen treatment. Scale bars: 100 μm. (**G**) Quantification of valve leaflet area from mutant and control hearts at days 4, 7, and 14 after tamoxifen treatment. *N* = 5 mice per group. **P < 0.01, ****P < 0.0001, by unpaired *t* tests. (**H**) Movat’s pentachrome staining of mitral and tricuspid valves from *Prox1^CreERT2+^* *Klf2^fl/fl^* *Klf4^fl/fl^* and *Klf2^fl/fl^* *Klf4^fl/fl^* mice at day 14 after tamoxifen treatment. (**I**) Quantification of valve proteoglycan area based on Alcian blue area in **H**. *P < 0.05, **P < 0.01, by 2-way ANOVA with Tukey’s multiple-comparison tests. LA, left atrium; LV, left ventricle; RA, right atrium. Arrows indicate flow side of the valve. Scale bars: 50 μm (**C** and **H**); 100 μm (**E**).

The above studies support a model in which hemodynamic forces maintain homeostasis in the adult valve, perhaps through expression of VEC KLF2/4. To directly test the role of endothelial KLF2/4 in mature cardiac valves, we used a tamoxifen-inducible Cre recombinase allele under control of the *Prox1* promoter (*Prox1^CreERT2^*) (16, 28, 29). Prox1 is induced by oscillatory fluid forces predicted to arise on the fibrosa (“non-flow”) side of the cardiac valves as well as in venous and lymphatic valves (30, 31). We crossed *Prox1^CreERT2+^* mice with mice carrying an *Ai14 RFP* reporter allele and assessed reporter activity throughout the heart following 5 days of tamoxifen gavage (32). Immunostaining for RFP and PECAM-1 revealed that reporter activity was restricted to the endothelium of cardiac valves and was not active in coronary vessel endothelium or ventricular endocardium (Figure 3A). It further revealed *Prox1^CreERT2^* activity in VECs on both the flow and non-flow sides of the cardiac valves (Figure 3A). *Prox1^CreERT2^* activity on both sides of the cardiac valve may be explained by the presence of some oscillatory flow on the flow side and the high activity of this BAC transgene.

We next crossed *Prox1^CreERT2+^* mice with mice carrying *Klf2*-floxed (*Klf2^fl/fl^*) and *Klf4*-floxed (*Klf4^fl/fl^*) alleles to generate *Prox1^CreERT2+^ Klf2^fl/fl^ Klf4^fl/fl^* animals in which *Klf2* and *Klf4* could be selectively deleted in cardiac VECs after administration of tamoxifen. Importantly, this inducible genetic system allows cardiac valves to develop normally. At 8–10 weeks of age, mice were given daily oral tamoxifen via gavage for 5 days, and the heart was harvested at days 4, 7, and 14 after the first tamoxifen dose (Figure 3B). We validated valve endothelium-specific deletion of KLF2 and KLF4 four days after tamoxifen treatment using a combination of immunohistochemistry for KLF4 (Figure 3, C–E) and in situ hybridization for *Klf2* (Supplemental Figure 3, A and B). Severely reduced *Klf2* and *Klf4* mRNA transcripts were identified in VECs using single-cell RNA sequencing (scRNA-Seq), in association with significant downregulation of known KLF2/4 targets *Nos3*, *Thbd*, and *Pi16* (Supplemental Figure 3, C–E, and below). H&E staining of hearts collected from *Prox1^CreERT2+^* *Klf2^fl/fl^* *Klf4^fl/fl^* animals demonstrated a progressive increase in the leaflet area of sectioned mitral and tricuspid valves compared with control valves between 4 and 14 days post-tamoxifen (Figure 3, F and G). A similar phenotype was seen in the aortic and pulmonic valves, in which valve leaflet area was also significantly increased (Supplemental Figure 4, A and B). To further assess valve changes following loss of KLF2 and KLF4, we performed Movat’s pentachrome staining to assess matrix GAG expression. As reported previously in human and mouse myxomatous valves, we observed an expansion of the proteoglycan content (indicated by blue staining) within the mitral and tricuspid valves in *Prox1^CreERT2+^* *Klf2^fl/fl^* *Klf4^fl/fl^* mice at 2 weeks post-tamoxifen (Figure 3H). Quantification of valve proteoglycan content revealed significant increases in both tricuspid and mitral valves following loss of VEC KLF2/4 (Figure 3I). Additionally, we found a significant increase in valve versican abundance in *Prox1^CreERT2+^* *Klf2^fl/fl^* *Klf4^fl/fl^* mice at 2 weeks post-tamoxifen (Supplemental Figure 5, A and B).

**Figure 4:**
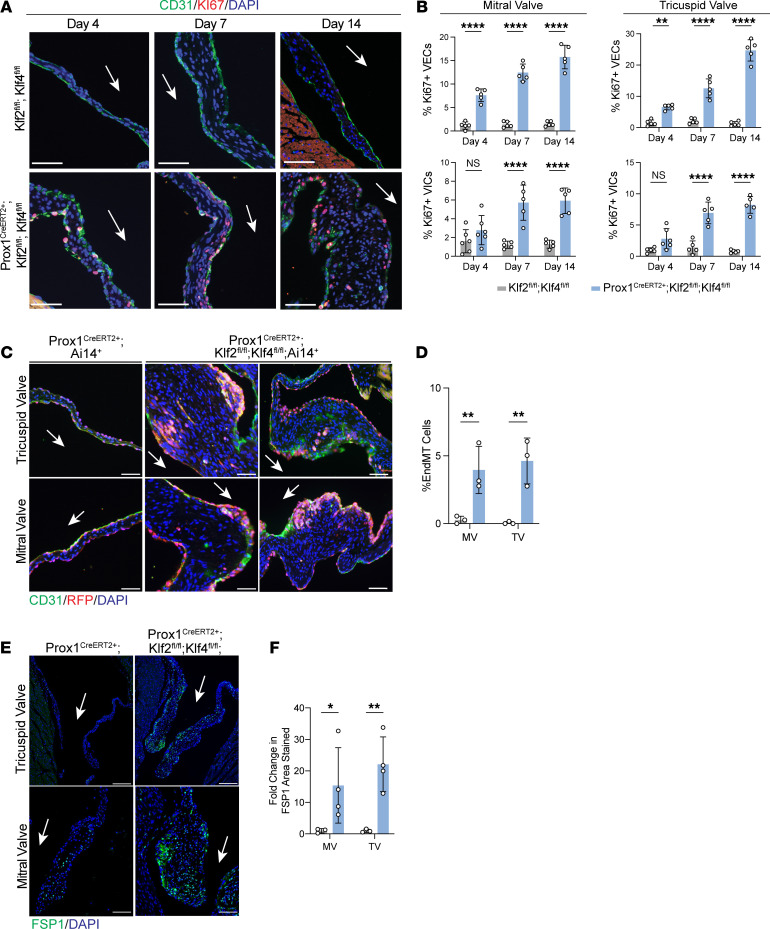
Loss of endothelial KLF2/4 in the mature heart valve leads to proliferation and endothelial-mesenchymal transition. (**A**) Immunostaining for PECAM-1, Ki67, and DAPI in tricuspid valve at days 4, 7, and 14 after tamoxifen treatment. (**B**) Quantification of Ki67^+^ cells in PECAM-1^+^ and PECAM-1^–^ cells. *N* = 5 mice per group. **P < 0.01, ****P < 0.0001, by unpaired *t* tests. (**C**) Immunostaining for RFP, PECAM-1, and DAPI 14 days after tamoxifen treatment. Scale bars: 50 μm (**A** and **C**). (**D**) Quantification of percentage endothelial-mesenchymal transition (EndMT) cells from **C**. **P < 0.01, by unpaired *t* tests. (**E**) Immunostaining for FSP1 (S100A4) and DAPI 14 days after tamoxifen treatment. Scale bars: 100 μm. Arrows indicate flow side of the valve. (**F**) Quantification of FSP1 staining in **E**. *P < 0.05, **P < 0.01, by unpaired *t* tests.

Given these observed histologic changes in valve morphology and proteoglycan expression, which are also characteristic of the human MVD phenotype, we further investigated cellular changes within the valves of *Prox1^CreERT2+^* *Klf2^fl/fl^* *Klf4^fl/fl^* mice. Immunostaining for PECAM-1 and Ki67 to assess valve cell proliferation revealed that the number of Ki67^+^PECAM-1^+^ VECs and the number of Ki67^+^PECAM-1^–^ VICs were both significantly increased at days 4, 7, and 14 post-tamoxifen (Figure 4, A and B). To lineage-trace VECs after loss of KLF2/4, we crossed in the Ai14 tdTomato reporter allele to *Prox1^CreERT2+^* *Klf2^fl/fl^* *Klf4^fl/fl^* mice. Immunostaining for RFP and CD31 revealed the presence of a small number of RFP^+^CD31^+^ cells within the interior of the valve leaflet as well as scattered clusters of lineage-positive cells just below the valve endothelium 14 days after tamoxifen treatment (Figure 4, C and D). These findings are suggestive of endothelial-mesenchymal transition (EndMT) following loss of KLF2/4, as has been previously reported (33, 34). Immunostaining also revealed elevated expression of fibroblast-specific protein-1 (FSP1; or S100A4), which showed increased expression in valves of *Prox1^CreERT2+^* *Klf2^fl/fl^* *Klf4^fl/fl^* mice (Figure 4, E and F). Echocardiographic studies failed to demonstrate mitral insufficiency when performed 14 days after tamoxifen induction (Supplemental Figure 6). In line with previous studies demonstrating compensatory increases in remaining *Klf2* and *Klf4* allele expression, deletion of either both alleles of *Klf2* and one allele of *Klf4* or both alleles of *Klf4* and one allele of *Klf2* showed no changes in valve size, even more than 1 year after deletion (Supplemental Figure 7) (20, 35). These findings closely mirror those observed following mechanical unloading with HHT and reveal that loss of either flow or the flow-responsive KLF2 and KLF4 transcription factors is sufficient to confer an MVD phenotype in mice.

### Single-cell RNA-Seq of Prox1^CreERT2+^ Klf2^fl/fl^ Klf4^fl/fl^ valves demonstrates increased monocyte recruitment and activation of TGF-β/Smad signaling.

**Figure 5:**
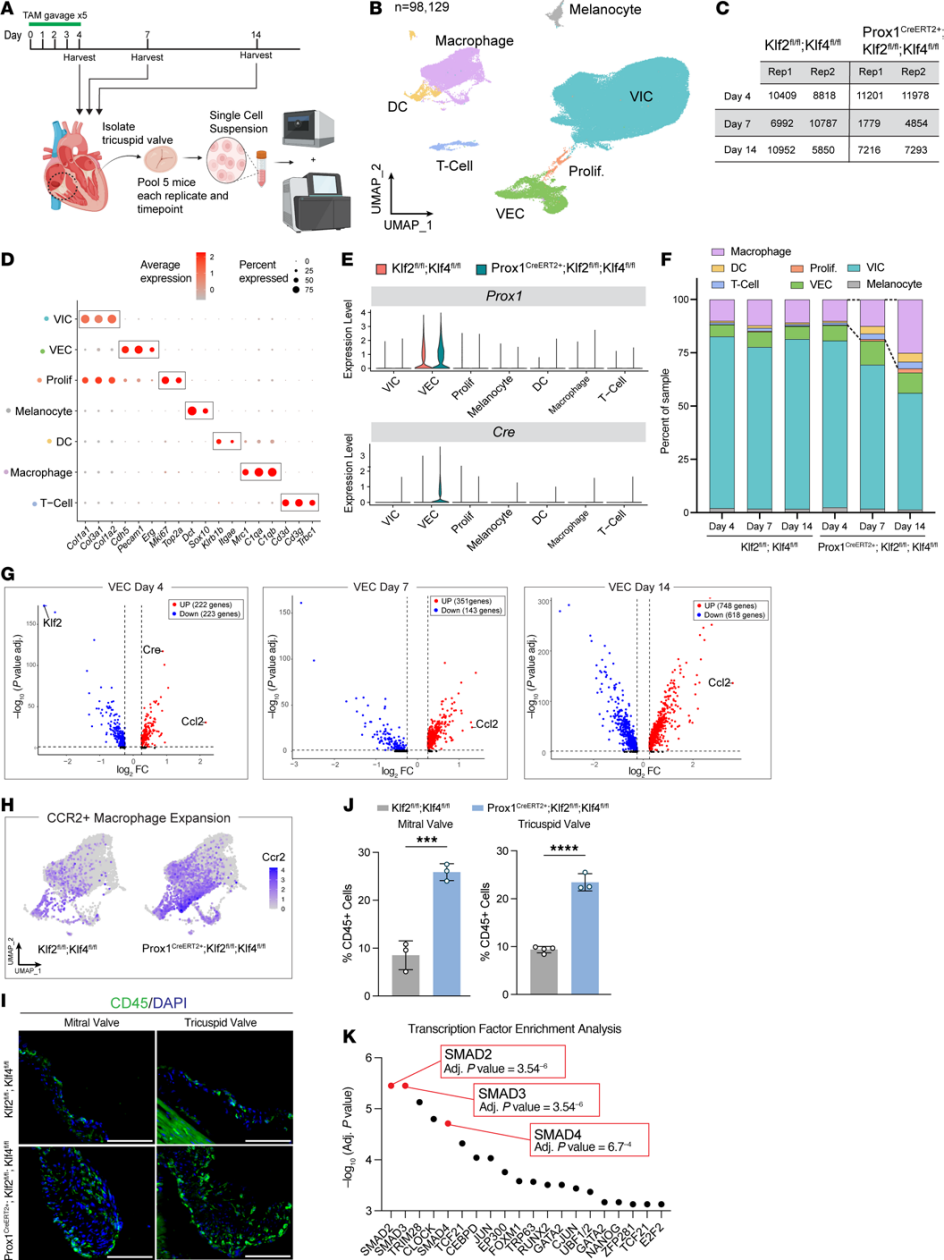
scRNA-Seq demonstrates monocyte recruitment and increased TGF-β/Smad signaling in myxomatous valves. (**A**) Tamoxifen-treated *Prox1^CreERT2+^ Klf2^fl/fl^ Klf4^fl/fl^* and *Klf2^fl/fl^ Klf4^fl/fl^* mice were sacrificed at days 4, 7, and 14. Tricuspid valves of 5–6 mice per sample and per time point were isolated and profiled using scRNA-Seq. (**B**) Uniform manifold approximation and projection (UMAP) plot illustrating 98,129 cells captured and sorted into 7 different clusters. (**C**) Number of cells captured at each time point and replicates that passed quality control filtering. (**D**) Dot plot showing marker genes enriched in each cluster used for cell type assignment. Color intensity is scaled to indicate average expression, and the point size indicates percentage of cells expressing the gene in the given cluster. (**E**) Violin plot highlighting expression of *Prox1* and *Cre* recombinase transcripts in VECs from *Prox1^CreERT2+^ Klf2^fl/fl^ Klf4^fl/fl^* mice. (**F**) Relative cell type abundance in *Prox1^CreERT2+^ Klf2^fl/fl^ Klf4^fl/fl^* and *Klf2^fl/fl^ Klf4^fl/fl^* mice at days 4, 7, and 14 post-tamoxifen. (**G**) Volcano plots showing differentially expressed genes within the VEC cluster at each time point. Genes in red are significantly upregulated, with a log_2_ fold change greater than 0.3 and an adjusted *P* value less than 0.05, and genes in blue represent significantly downregulated genes, with a log_2_ fold change less than –0.3 and an adjusted *P* value less than 0.05. (**H**) Feature plot of CCR2 expression in *Prox1^CreERT2+^ Klf2^fl/fl^ Klf4^fl/fl^* and *Klf2^fl/fl^ Klf4^fl/fl^* mice. (**I**) Immunostaining for CD45 in mitral and tricuspid valves 14 days after tamoxifen treatment in the indicated animals. Scale bars: 50 μm. (**J**) Quantification of percentage of CD45^+^ cells in both mitral and tricuspid valves. *N* = 3 mice per group. ***P < 0.001, ****P < 0.0001, by unpaired *t* tests. (**K**) Transcription factor enrichment analysis plot of top upregulated genes in *Prox1^CreERT2+^ Klf2^fl/fl^ Klf4^fl/fl^* VECs.

Cardiac valves are composed of several cell types, including interstitial cells, endothelial cells, and immune cells. Furthermore, these broad cell types exist as heterogeneous populations, e.g., endothelial cells on different sides of the valve that display distinct gene expression patterns. To understand how loss of KLF2/4 gene regulation in VECs confers a myxomatous valve phenotype, we isolated tricuspid valves from *Prox1^CreERT2+^* *Klf2^fl/fl^* *Klf4^fl/fl^* and control mice at days 4, 7, and 14 post-tamoxifen and performed scRNA-Seq. For these studies we pooled valve tissue from 5 mice for each genotype at each time point in 2 replicate pools and performed genome-wide scRNA-Seq (Figure 5A). After standard quality control and filtering steps, we generated transcriptomic profiles of 98,129 cells across both genotypes and all 3 time points (Figure 5, A–C). Unsupervised clustering analysis yielded discrete groupings of the main cell types expected in the mature murine tricuspid valve, including VECs, VICs, T cells, dendritic cells, macrophages, and melanocytes (Figure 5B). Cluster identities were annotated based on an assortment of significantly enriched marker genes (Figure 5D). Interestingly, we identified a novel cluster of proliferating cells, which was enriched for both valve interstitial and endothelial marker genes, along with proliferation markers including *Mki67* and *Top2a* (Figure 5D). Further analysis of this cluster showed that these proliferating cells expressed either endothelial genes or VIC genes, but not both (Supplemental Figure 8). Significantly, expression of *Cre* recombinase transcript was highly specific to the VEC population of *Prox1^CreERT2+^* *Klf2^fl/fl^* *Klf4^fl/fl^* mice, which also expresses *Prox1*, and was not significantly detected in either the VIC or the immune cell cluster (Figure 5E).

After analyzing the relative cell type abundances in each sample at these time points, we observed a notable increase in the fraction of immune cells present in *Prox1^CreERT2+^* *Klf2^fl/fl^* *Klf4^fl/fl^* valves compared with controls after day 4 (Figure 5F). We hypothesized that an endothelium-derived cytokine may be responsible for increased recruitment of immune cells to the valve leaflets, and that subsequent inflammatory activity might confer MVD changes. Differential gene expression testing to identify genes that were up- or downregulated specifically in VECs of *Prox1^CreERT2+^* *Klf2^fl/fl^* *Klf4^fl/fl^* mice at each time point identified C-C motif chemokine ligand 2 (*Ccl2*) as the most highly upregulated gene, with activation as early as day 4 post-tamoxifen and maintained through days 7 and 14 (Figure 5G). CCL2, also referred to as MCP-1, signals through C-C chemokine receptor 2 (CCR2) to recruit and activate circulating monocytes (36), and has recently been implicated in MVD conferred by Marfan disease (37). We queried the expression of *Ccr2* in the macrophage populations we identified in scRNA-Seq and found that there was marked expansion of CCR2^+^ macrophages in *Prox1^CreERT2+^* *Klf2^fl/fl^* *Klf4^fl/fl^* valves compared with controls (Figure 5H). Immunostaining for the hematopoietic cell marker CD45 in valve leaflets revealed a significant increase in CD45^+^ cells in both mitral and tricuspid valves of *Prox1^CreERT2+^* *Klf2^fl/fl^* *Klf4^fl/fl^* mice compared with controls (Figure 5, I and J). To identify potential upstream factors regulating the changes observed in VECs, including upregulation of *Ccl2*, we performed transcription factor enrichment analysis using the top 50 significantly differentially expressed genes in the VEC cluster following loss of KLF2/4. This analysis identified Smads 2, 3, and 4 among the top enriched transcription factors, suggesting that activated TGF-β/Smad signaling may contribute to the transcriptional changes observed and the MVD phenotype (Figure 5K and Supplemental Figure 9).

### Loss of Ccr2 prevents monocyte recruitment but not MVD in Prox1^CreERT2+^ Klf2^fl/fl^ Klf4^fl/fl^ mice.

Increased immune cell recruitment to myxomatous valves has been recently identified in the *Fbn1^C1039G/+^* mouse model of Marfan syndrome, with deficiency in circulating monocytes reported to attenuate the myxomatous phenotype (6, 37). Since *Ccl2* was among the top differentially expressed genes in VECs of *Prox1^CreERT2+^* *Klf2^fl/fl^* *Klf4^fl/fl^* mice, we sought to determine whether blocking monocyte recruitment to the valve via reduced CCL2/CCR2 signaling could modulate the myxomatous phenotype in *Prox1^CreERT2+^* *Klf2^fl/fl^* *Klf4^fl/fl^* valves.

To test the role of CCL2/CCR2 signaling, we first used a bone marrow (BM) transplantation approach. We irradiated recipient *Klf2^fl/fl^* *Klf4^fl/fl^* and *Prox1^CreERT2+^* *Klf2^fl/fl^* *Klf4^fl/fl^* mice, which were then reconstituted with BM isolated from *Ccr2^+/GFP^* or *Ccr2^GFP/GFP^* mice, which harbor a GFP allele that replaces the *Ccr2* coding region (38). After BM transplantation, mice received 5 days of tamoxifen, and hearts were collected for histologic analysis after 14 days. H&E staining to assess for change in valve thickening revealed similar marked increases in valve thickness and area in both *Prox1^CreERT2+^* *Klf2^fl/fl^* *Klf4^fl/fl^+Ccr2^GFP/GFP^* BM mice and *Prox1^CreERT2+^* *Klf2^fl/fl^* *Klf4^fl/fl^+Ccr2^+/GFP^* BM mice (Supplemental Figure 10, A and B), suggesting that loss of CCL2/CCR2 signaling is not sufficient to rescue the MVD phenotype conferred by loss of KLF2/4 function in VECs.

Since irradiation required for BM transplantation could also impact cellular responses in the cardiac valves, to further test the role of CCL2/CCR2 signaling we used a genetic model in which we crossed *Prox1^CreERT2+^ Klf2^fl/fl^* *Klf4^fl/fl^* mice with *Ccr2^GFP/GFP^* mice to generate *Prox1^CreERT2+^* *Klf2^fl/fl^* *Klf4^fl/fl^* *Ccr2^GFP/GFP^* and littermate control animals. Quantification of both mitral and tricuspid valve area 14 days after tamoxifen-induced deletion of VEC *Klf2* and *Klf4* demonstrated increased valve area in *Prox1^CreERT2+^* *Klf2^fl/fl^* *Klf4^fl/fl^* *Ccr2^GFP/GFP^* valves that was comparable to that in both *Prox1^CreERT2+^* *Klf2^fl/fl^* *Klf4^fl/fl^* *Ccr2^GFP/+^* and *Prox1^CreERT2+^* *Klf2^fl/fl^* *Klf4^fl/fl^* *Ccr2^+/+^* control valves (Supplemental Figure 11, A–C). Immunostaining of valve tissue sections for GFP confirmed that the number of GFP^+^ cells in the valve leaflet was highly reduced following loss of CCR2 even though they remained myxomatous (Supplemental Figure 11, D–F). Immunostaining for CD45 confirmed these findings, with markedly reduced numbers of CD45^+^ hematopoietic cells detected in myxomatous valves following loss of CCR2 (Supplemental Figure 11, G–I). These results are consistent with the BM transplantation studies and support the conclusion that myxomatous valve changes in *Prox1^CreERT2+^* *Klf2^fl/fl^* *Klf4^fl/fl^* mice arise in a manner that is independent of CCR2.

### Loss of Ccr2 does not prevent MVD in Fbn1-mutant mice.

The myxomatous valve phenotype of *Prox1^CreERT2+^* *Klf2^fl/fl^* *Klf4^fl/fl^* mice is very similar to that of the well-established Marfan syndrome mouse model *Fbn1^C1039G/+^* (7). However, the myxomatous valve phenotype in *Fbn1^C1039G/+^* mice was recently reported to be reversed by CCR2 loss, while that in *Prox1^CreERT2+^* *Klf2^fl/fl^* *Klf4^fl/fl^* mice was not (37). These observations suggested either that myxomatous valve phenotypes could arise through distinct pathogenic mechanisms despite sharing many characteristic phenotypes — including recruitment of CCR2^+^ monocytes — or that the role of CCL2/CCR2 signaling is not causal as previously reported. To distinguish between these possibilities, we repeated the studies reported to demonstrate rescue of myxomatous valve pathology in *Fbn1^C1039G/+^* mice with loss of CCR2 (37). We crossed *Ccr2^GFP/GFP^* mice and *Fbn1^C1039G/+^* mice to generate *Fbn1^C1039G/+^ Ccr2^GFP/GFP^* mice and *Fbn1^C1039G/+^* *Ccr2^GFP/+^* control littermates. As previously reported (7, 37), *Fbn1^C1039G/+^* *Ccr2^GFP/+^* mice displayed characteristic myxomatous mitral and tricuspid valves by 2 months of age (Supplemental Figure 12, A–C), and loss of CCR2 conferred complete loss of CCR2^+^ macrophages in the *Fbn1^C1039G/+^* myxomatous valve (Supplemental Figure 12, D–I). However, quantitative analysis demonstrated increased valve leaflet size in *Fbn1^C1039G/+^* *Ccr2^GFP/GFP^* valves lacking CCR^+^ macrophages that was indistinguishable from that observed in *Fbn1^C1039G/+^* *Ccr2^+/GFP^* littermate valves at 2 months of age (Supplemental Figure 12, A–C). These findings demonstrate that recruitment of CCR2^+^ monocytes is a prominent feature of MVD conferred by both expression of mutant FBN1 and VEC loss of KLF2/4, but that in neither case is this recruitment required for myxomatous valve formation. Instead, our findings suggested an important role for a distinct, valve-intrinsic mechanism for MVD present in both models.

### Activation of TGF-β/SMAD signaling drives MVD in Prox1^CreERT2+^ Klf2^fl/fl^ Klf4^fl/fl^ valves.

**Figure 6:**
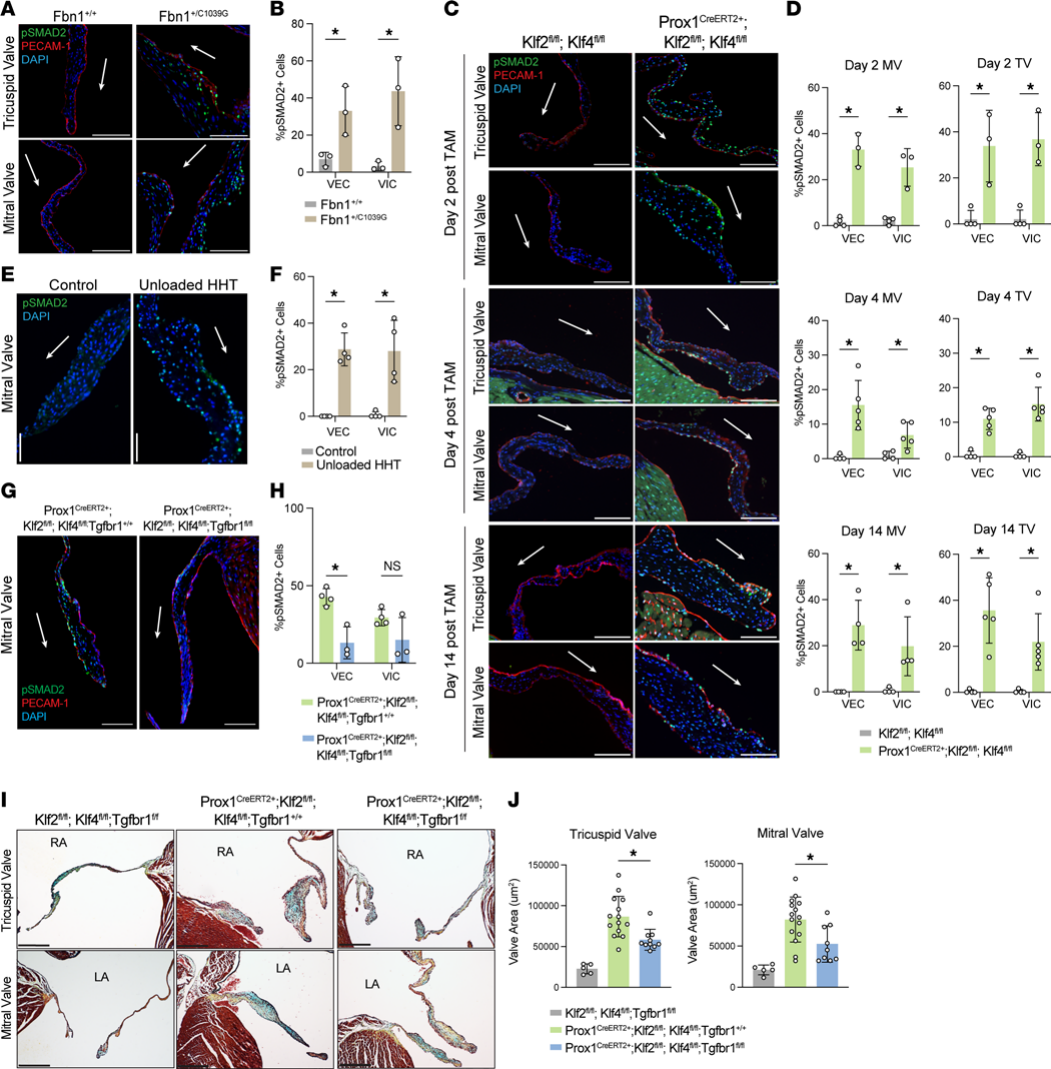
Increased p-SMAD2 in myxomatous valves from *Prox1^CreERT2+^ Klf2^fl/fl^ Klf4^fl/fl^* and *Fbn1*^C1039G/+^ mice. (**A**) Immunostaining for p-SMAD2 and PECAM-1 with DAPI in tricuspid and mitral valve of *Fbn1^C1039G/+^* mice and controls at 1 month of age. Scale bars: 100 μm. (**B**) Quantification of VEC and VIC p-SMAD2 in **A**. *N* ≥ 4 mice per group. *P < 0.05, by unpaired *t* tests. (**C**) Immunostaining for p-SMAD2 and PECAM-1 with DAPI in tricuspid and mitral valve of *Prox1^CreERT2+^ Klf2^fl/fl^ Klf4^fl/fl^* mice and controls 2 and 4 days after the first dose of tamoxifen. Scale bars: 100 μm. (**D**) Quantification of VEC and VIC p-SMAD2 in **C**. *N* ≥ 4 mice per group. *P < 0.05, by unpaired *t* tests. (**E**) Immunostaining for p-SMAD2 with DAPI in mitral valve of unloaded HHT mice and controls at day 4 after transplant. Scale bars: 50 μm. (**F**) Quantification of VEC and VIC p-SMAD2 in **E**. *N* = 4 mice per group. *P < 0.05, by unpaired *t* tests. (**G**) Immunostaining for p-SMAD2 and PECAM-1 with DAPI in mitral valve of *Prox1^CreERT2+^ Klf2^fl/fl^ Klf4^fl/fl^ Tgfbr1^+/+^* and *Prox1^CreERT2+^ Klf2^fl/fl^ Klf4^fl/fl^ Tgfbr1^fl/fl^* mice 4 days after tamoxifen. Scale bars: 50 μm. (**H**) Quantification of VEC and VIC p-SMAD2 in **G**. *N* ≥ 3 mice per group. *P < 0.05, by unpaired *t* tests. (**I**) H&E staining of mitral and tricuspid valves of *Prox1^CreERT2+^ Klf2^fl/fl^ Klf4^fl/fl^ Tgfbr1^+/+^* and *Prox1^CreERT2+^ Klf2^fl/fl^ Klf4^fl/fl^ Tgfbr1^fl/fl^* mice at day 14 after tamoxifen. Scale bars: 50 μm. (**J**) Quantification of valve leaflet area in **I**. *N* ≥ 5 mice per group. *P < 0.05, by 1-way ANOVA with Tukey’s multiple-comparison test. Arrows indicate flow side of the valve.

The studies described above suggested that MVD due to either VEC loss of KLF2/4 activity or mutation of the extracellular matrix protein FBN1 may arise through a common molecular mechanism other than monocyte recruitment. Activated TGF-β/SMAD signaling has been implicated in the pathophysiology of myxomatous valve formation in both human Marfan syndrome and the *Fbn1^C1039G/+^* Marfan mouse model (3, 7, 8, 39), and has also been recently associated with loss of endothelial MEKK3-KLF2/4 signaling in the vasculature (33). Since analysis of scRNA-Seq data suggested increased SMAD activity in myxomatous *Prox1^CreERT2+^* *Klf2^fl/fl^* *Klf4^fl/fl^* valves, we next assessed TGF-β/Smad signaling as an underlying common mechanism for MVD. Consistent with prior reports, immunostaining for phospho-SMAD2 (p-SMAD2), a marker of active TGF-β signaling, and PECAM-1 revealed elevated p-SMAD2 in both VICs and VECs in the valves of *Fbn1^C1039G/+^* mice by 1 month of age (Figure 6, A and B). In the *Fbn1^C1039G/+^* mouse Marfan syndrome model, expression of valve endothelial *Klf2*/*4* was unchanged in comparison with control tissue (Supplemental Figure 13). Staining of valves from *Prox1^CreERT2+^* *Klf2^fl/fl^* *Klf4^fl/fl^* animals also revealed strong p-SMAD2 starting as early as 2 days after the first dose of tamoxifen (Figure 6, C and D). Remarkably, p-SMAD2 was observed at this very early time point at similar levels in both VECs and VICs despite restriction of CreERT2 activity and gene deletion to VECs in *Prox1^CreERT2+^* *Klf2^fl/fl^* *Klf4^fl/fl^* animals. These results are consistent with a rapid, non-cell-autonomous effect of endothelial KLF2/4 loss like that associated with FBN1 mutation. Further analysis of the scRNA-Seq data revealed similar enrichment of TGF-β/Smad signaling activation in both VECs and VICs, based on calculation of a SMAD target gene enrichment and cell-cell communication analysis (Supplemental Figure 14). Consistent with the tight correlation of histologic and molecular findings following mechanical valve unloading with HHT and genetic deletion of KLF2/4 function in VECs with Prox1-CreERT2, increased p-SMAD2 was also detected in the unloaded HHT donor heart at day 4 (Figure 6, E and F). These findings suggested that loss of hemodynamic shear and KLF2/4 function might confer a myxomatous valve phenotype by stimulating valvular TGF-β signaling. Although numerous type 1 and type 2 receptors may participate in TGF-β signaling, TGF-βR1 (also called ALK5) is a commonly utilized receptor that has recently been shown to mediate upregulated endothelial TGF-β signaling following loss of MEKK3, an upstream regulator of KLF2/4 expression, in the pulmonary vascular endothelium (33). To test a causal role for TGF-β signaling, we therefore generated *Prox1^CreERT2+^* *Klf2^fl/fl^* *Klf4^fl/fl^* *Tgfbr1^fl/fl^* mice and control littermates in which VEC loss of KLF2/4 function was accompanied by VEC loss of TGF-βR1. Concomitant loss of TGF-βR1 reversed the increase in p-SMAD2 staining in VECs lacking KLF2/4 (Figure 6, G and H) and partially corrected the myxomatous phenotype and increased valve area (Figure 6, G–J). The inability of this strategy to fully rescue the myxomatous phenotype may reflect its inability to block TGF-β signaling in VICs as well as VECs (discussed further below). These findings implicate augmented TGF-β signaling as a common mechanism by which changes in either hemodynamic shear and KLF2/4 signaling or extracellular matrix and FBN function may confer MVD.

### Reduced KLF2/4 expression and increased phospho-SMAD2 in human MVD.

**Figure 7:**
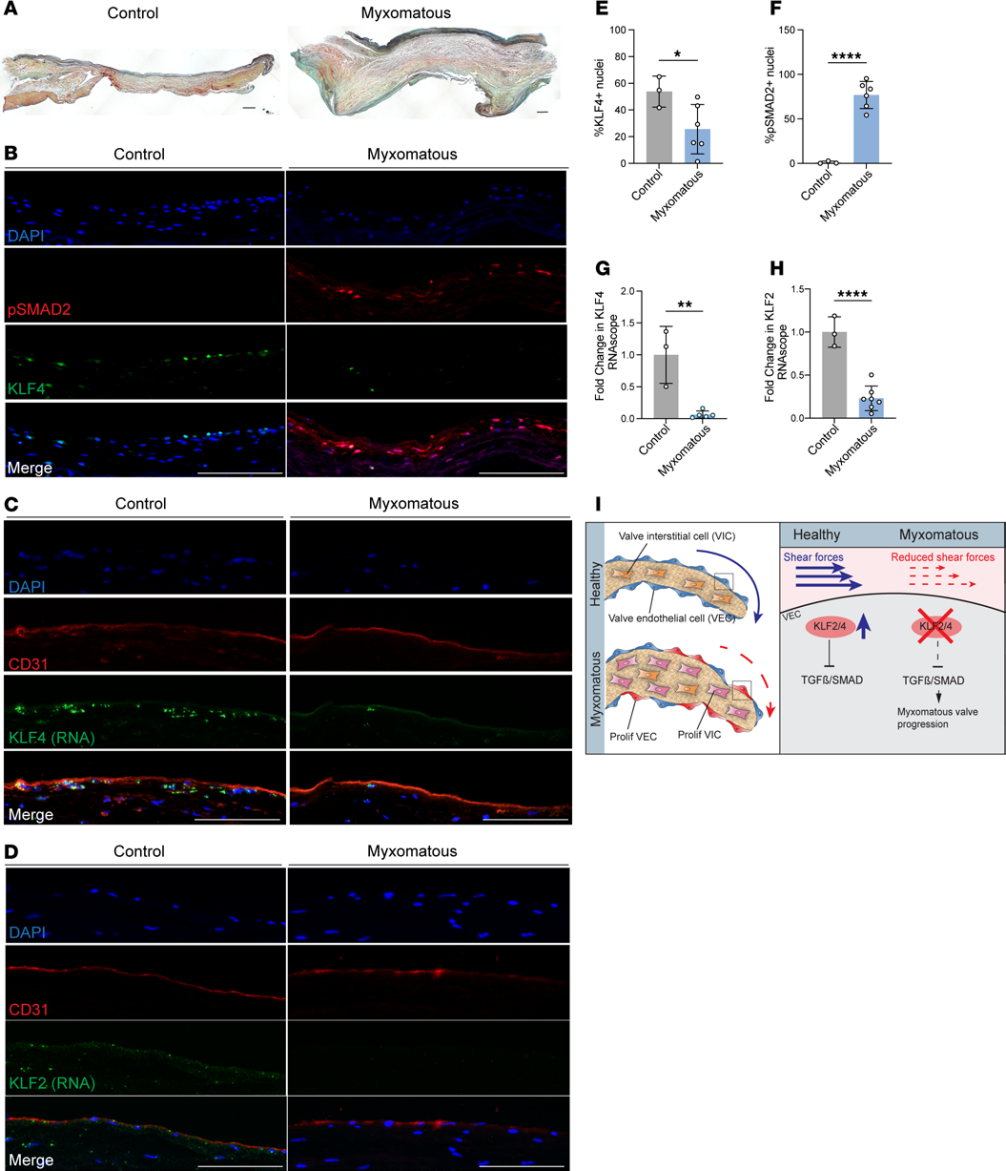
KLF2/4 and p-SMAD2 are altered in human myxomatous mitral valves. (**A**) Movat’s pentachrome staining of human myxomatous mitral valves and non-myxomatous mitral valve controls. Scale bars: 200 μm. (**B**) Immunostaining for p-SMAD2 and KLF4. Scale bars: 100 μm. (**C**) In situ hybridization for *KLF4* mRNA and costaining with CD31. Scale bars: 100 μm. (**D**) In situ hybridization for *KLF2* mRNA and costaining with CD31. Scale bars: 100 μm. (**E**) Quantification of KLF4^+^ nuclei in **B**. *N* ≥ 3 samples per group. *P < 0.05, by unpaired *t* tests. (**F**) Quantification of p-SMAD2^+^ nuclei in **B**. *N* ≥ 3 samples per group. ****P < 0.0001, by unpaired *t* tests. (**G**) Quantification of *KLF4* mRNA in **C**. *N* ≥ 3 samples per group. **P < 0.01, by unpaired *t* tests. (**H**) Quantification of *KLF2* mRNA in **C**. *N* ≥ 3 samples per group. ****P < 0.0001, by unpaired *t* tests. (**I**) Schematic of proposed mechanism of myxomatous valve formation following loss of KLF2/4 either by genetic deletion or loss when the valve is exposed to reduced flow.

Our mouse genetic studies suggested that altered hemodynamic forces associated with loss of valve endothelial KLF2/4 expression are sufficient to augment TGF-β–SMAD2/3 signaling and confer MVD. To determine whether non-syndromic human MVD might be consistent with this mechanism, we next assessed mitral valves harvested from control human hearts and from those with MVD. As reported previously, the myxomatous human mitral valve exhibited thickening associated with increased matrix deposition in the valve interstitium (Figure 7A). Immunostaining revealed reduced numbers of KLF4^+^ nuclei and increased numbers of phospho-SMAD2^+^ nuclei in the human myxomatous valve (Figure 7, B, E, and F). RNAscope performed to detect *KLF4* and *KLF2* mRNA also revealed reduced *KLF2 and KLF4* expression in the myxomatous mitral valve relative to control (Figure 7, C, D, G, and H). These findings in human myxomatous valves are consistent with those obtained using the mouse physiologic and genetic models described above and support a model in which altered hemodynamic forces across valvular endothelium can confer MVD (Figure 7I and discussed below).

## Discussion

The cardiac valves are exposed to constant hemodynamic and mechanical stimuli. Previous studies have begun to elucidate the pathways that link hemodynamic forces to valve development, but the role of such forces and pathways in the adult cardiac valve has remained uninvestigated. Most MVD arises in older individuals who lack genetic defects associated with syndromic valvular heart disease. Thus, environmental factors and “wear and tear” mechanisms associated with aging appear to play a prominent role in MVD pathogenesis, but these remain to be identified at the molecular and cellular levels. In the present study, we use a combination of genetic and surgical approaches to interrogate the role of blood flow and the key endothelial transcription factors KLF2 and KLF4 in maintenance of valve homeostasis. Our findings demonstrate that hemodynamic forces associated with flowing blood prevent myxomatous changes in cardiac valves, and that this homeostatic function requires flow-induced expression of the valve endothelial KLF2 and KLF4 transcription factors. Transcriptional profiling and genetic rescue studies identify suppression of TGF-β signaling, but not valvular monocyte recruitment, as a causal mechanism underlying MVD associated with loss of KLF2/4 function. These studies identify a potential molecular mechanism for acquired MVD, extend our understanding of the homeostatic roles of blood flow and endothelial KLF2/4 function in the mature cardiovascular system, and suggest that excess TGF-β signaling may underlie both syndromic and non-syndromic MVD.

Studies performed over the past two decades have established that high laminar shear forces confer a quiescent, non-inflammatory state in the arterial vasculature that prevents acquired diseases such as atherosclerosis (26, 40). Much of this effect has been attributed to high expression of the endothelial transcription factors KLF2 and KLF4 (15, 25). Our findings extend this paradigm and suggest that hemodynamic shear forces and endothelial KLF2/4 also play a homeostatic role in cardiac valves, and that acquired MVD may be linked to loss of this protective effect. How might MVD arise as a result of altered valve hemodynamic forces? It is possible that small imperfections in the surface of a cardiac valve, e.g., due to a minor developmental defect or as a consequence of daily “wear and tear,” might result in uneven hemodynamic shear forces across the valvular endothelium and foci of reduced KLF2/4 activity in which myxomatous changes (i.e., VEC and VIC proliferation, matrix deposition, and monocyte recruitment) arise. Once present, such defects might perpetuate heterogeneity of valvular shear forces and promote further myxomatous responses (Figure 7I). As the only bicuspid valve with the largest leaflets, the mitral valve might be particularly prone to such a hemodynamic imperfection mechanism. Analysis of human myxomatous mitral valve leaflets revealed decreased endothelial KLF2/4 expression and increases in valvular p-SMAD2, suggesting that such a mechanism may underlie human MVD.

Our studies identify gain of TGF-β signaling as an important downstream effector by which loss of KLF2/4 function confers MVD in mice. These findings are consistent with prior studies of KLF2/4-deficient vascular inflammatory responses. In vitro studies have demonstrated that loss of KLF2/4 augments expression of TGF-β–inducible genes and that shear forces suppress SMAD2/3 nuclear translocation through MEKK3-KLF2/4 signaling (33, 41–44). In vivo studies have associated loss of MEKK3-KLF2/4 signaling with activation of TGF-β signaling and arterial remodeling that was also TGF-βR1 dependent (33). Thus, loss of laminar shear responses mediated by KLF2/4 appears to drive both vascular and valvular changes in part through TGF-β signaling. CDK2-mediated phosphorylation of SMAD proteins has been proposed as a mechanism for augmented TGF-β signaling following loss of MEKK3-KLF2/4 signaling (43), but this would confer primarily a cell-autonomous change in SMAD signaling, while we observed early and simultaneous elevation of p-SMAD activity in both VECs and VICs (Figure 6C). Simultaneous activation of TGF-β signaling in VECs and VICs suggests that increased TGF-β signaling may be mediated by changes in the extracellular matrix environment, a known key regulator of TGF-β ligand availability and activity (45, 46). Analysis of changes in the expression of VEC-secreted proteins following loss of KLF2/4 revealed high levels of CCL2 (discussed below) but relatively small, although significant, increases in potential extracellular TGF-β activators including thrombospondin-1 and Adamts1 (47, 48). Thus, future studies are needed to better define how KLF2/4 function controls TGF-β signaling to maintain valvular homeostasis.

Our finding that loss of KLF2/4 function occasionally induces endothelial-mesenchymal transition (EndMT) in VECs is consistent with published studies examining loss of the MEKK3-KLF2/4 signaling pathway in endothelial cells in other locations (33, 34, 44), but stands in contrast to a study reporting strain-dependent failure of EndMT in the KLF2-deficient cardiac cushion during valve development (49). In a prior study we did not observe a failure of EndMT in the developing valve cushion following endocardial loss of *Klf2*, or following loss of *Klf2* and one allele of *Klf4*, but instead noted a failure of cushion remodeling due to loss of KLF2/4-regulated Wnt9b expression (19). It is possible that this discrepancy in the role of KLF2/4 during EndMT during development is strain dependent. However, the postnatal studies appear concordant and demonstrate a gain of TGF-β signaling and EndMT as a common outcome after endothelial loss of homeostatic KLF2/4 function in numerous vascular environments. Thus it appears likely that such responses may also participate in human vascular and valvular pathologies.

The finding that KLF2/4 function prevents MVD by restraining TGF-β signaling is remarkable in light of human and mouse MVD conferred by mutations in FBN1 associated with Marfan syndrome, as well as increased TGF-β/Smad signaling observed in human and canine myxomatous valves that arise in the absence of known Marfan syndrome mutations (7, 12, 13, 37, 50–54). These findings are concordant and support elevated TGF-β/SMAD signaling as a common underlying mechanism for myxomatous valve formation. In addition to elevated TGF-β signaling, we observed elevated expression of the CCL2 chemokine by KLF2/4-deficient VECs and a sizable influx of CCR2^+^ monocytes into the myxomatous valve. Recruitment of CCR2^+^ monocytes was also recently observed in the *Fbn1^C1039G/+^* Marfan MVD mouse model, where it was further reported that loss of CCR2 prevented myxomatous changes (37). However, we found that concurrent loss of CCR2 failed to prevent formation of myxomatous valves, despite preventing recruitment of blood-borne monocytes, in both the KLF2/4 and Marfan models. These findings and the fact that elevated CCL2 expression is shared by both models suggest that CCL2-CCR2 monocyte recruitment is more likely to be a consequence than a cause of myxomatous changes, perhaps secondary to elevated TGF-β signaling. Our data are consistent with TGF-β/Smad signaling as a point of convergence between altered FBN1 and loss of VEC KLF2/4 in myxomatous valve degeneration. Based on published RNA-Seq data, VEC expression of *Klf2* and *Klf4* is unchanged in *Fbn1^C1039G/+^* mice, and expression of *Fbn1* is unchanged in our analysis of KLF2/4-deficient VECs. Thus the phenotypic convergence of these two pathways is more likely to take place at the level of TGF-β/Smad signaling (Supplemental Figure 13). Since other factors such as the regulation of endothelial cell junctions that may control monocyte recruitment have also recently been associated with MVD (27), the pathogenesis of this disease is likely to incorporate numerous downstream events.

### Limitations of the present study.

In this study we tested the role of hemodynamic forces using the HHT model. As discussed above, this model is associated with transient myocardial ischemia and reduced cardiac chamber size. We used a partially loaded HHT model to control for these effects, but cardiac filling is still lower than normal in this model. A significant limitation for analysis of the long-term effects of KLF2/4 loss in valvular endothelium is the fact that *Prox1^CreERT2+^* *Klf2^fl/fl^* *Klf4^fl/fl^* animals exhibit lymphatic phenotypes after 14 days that necessitate euthanasia. Thus, longer-term effects such as valvular regurgitation could not be examined in these animals.

## Methods

Further information can be found in Supplemental Methods.

### Sex as a biological variable.

Our studies involved use of both male and female mice, and sex was not considered as a biological variable.

### Mouse models.

*Klf2^fl^* (16), *Klf4^fl^* (29), *Tgfbr1^fl^* (55), *Fbn1^C1039G/+^* (28), *Ccr2^GFP^* (38), and *Ai14* (32) animals have been previously described and in some cases were obtained from The Jackson Laboratory. The *Prox1^CreERT2^* was provided by Tajia Makinen. All animals were housed in a pathogen-free environment in an Association for Assessment and Accreditation of Laboratory Animal Care International–approved (AAALAC-approved) vivarium at the University of Pennsylvania, and experiments were performed in accordance with the guidelines of the Committee for Animal Research. Tamoxifen was administered via oral gavage (5 mg/200 μL in corn oil per mouse).

### Heterotopic heart transplantation.

Heterotopic heart transplant studies were performed as described previously (22). Female C57BL/6J mice at 13 weeks of age were used for all transplantation procedures. For collection of donor hearts, mice were anesthetized using a single dose of pentobarbital (65 mg/kg, i.p.). The heart was surgically removed, flushed with chilled saline, and stored in chilled saline at 4°C until implantation into the recipient mouse. Recipient mice received sustained-release buprenorphine (1 mg/kg s.c.) and bupivacaine (2 mg/kg, s.c.) before surgery and were anesthetized using pentobarbital (65 mg/kg, i.p.). Recipients underwent a midline laparotomy, and the infrarenal vena cava and aorta were dissected and clamped with a bulldog clamp. For the unloaded transplant configuration, the donor aorta was sutured to the recipient abdominal aorta, and the donor pulmonary artery was sutured to the recipient inferior vena cava. For the loaded configuration, the donor superior vena cava was sutured to the recipient inferior vena cava, and the donor pulmonary artery was sutured to the donor left atrium. The clamp was released, and the donor heart began to beat spontaneously. The abdominal wall and skin were closed, forming a 2-layer close.

### Single-cell isolation.

For single-cell sequencing experiments, mice were perfused with cold PBS, and cardiac valves were harvested. Valve tissue was pooled from 5 mice for each group and kept in PBS on ice until ready for dissociation. Single-cell suspension was generated by incubation of valves in digestion buffer containing collagenase/dispase (Roche, 11097113001) and DNase I (Roche, 10104159001) at 37°C with rocking for 10 minutes. Valves were then mechanically dissociated by gentle pipetting 15 times and incubated for an additional 10 minutes at 37°C. Valves were gently pipetted 15 times again, and supernatant was mixed in isolation buffer containing 1% FBS and 2 mM EDTA. Cells were spun at 500 *g* for 4 minutes and resuspended in fresh isolation buffer on ice. Cells were filtered through a 40 μm filter, counted using a hemocytometer, and loaded into the 10x Genomics platform according to the manufacturer’s instructions with a target of 10,000 cells per sample.

### Human mitral valve specimens.

All human subjects research in this study, including the use of human tissues, conformed to the principles outlined in the Declaration of Helsinki. All patient information was deidentified. Exclusion criteria for this study included endocarditis, rheumatic heart disease, ischemic mitral regurgitation, a history of cancer, autoimmune diseases, previous mitral surgery, and any history of cardiac trauma. Patients with mitral regurgitation referred for first-time surgery at Columbia University were enrolled in this study. Informed consent was obtained per IRB (AAAR6796) upon admission prior to surgery. Normal mitral valve tissue was obtained from patients undergoing cardiac transplant with no MVD or from healthy hearts from cardiac donors that were allocated for cardiac transplant but ultimately not transplanted for logistical reasons.

### Histology and immunostaining.

Mice were perfused with 4% paraformaldehyde (Fisher, 50-980-495) diluted in 1× PBS and fixed in 4% paraformaldehyde overnight at 4°C before undergoing dehydration and paraffin embedding. Sections were dewaxed with xylenes and rehydrated with decreasing concentrations of ethanol before staining with H&E (Abcam, ab245880) or Movat’s pentachrome (Abcam, ab245884). For immunostaining, antigen retrieval was performed using IHC-Tek Epitope retrieval solution (IHC World, IW-1100) and blocking with 10% normal donkey serum and 1% BSA before primary antibody incubation at 4°C overnight. Fluorescence-conjugated Alexa Fluor secondary antibodies were used according to primary antibody species (1:500) along with Hoechst (1:1,000). For p-SMAD2 staining, ImmPress TSA-based amplification was used at a dilution of 1:100 for 6 minutes (Akoya Bioscience). All sections were mounted with ProLong Gold Antifade mounting medium (Thermo Fisher Scientific, P36930), and imaging was performed on an Olympus BX53 Microscope. Quantification of microscopy images was performed using ImageJ v2.0 (NIH), including calculation of valve leaflet area and percentage area stained and counting of cells. For quantification of valve leaflet area, the anterior and posterior leaflet areas from at least 6 sections per valve were averaged to produce a single data point for each mouse. RNAscope signal was quantified by measurement of percentage area stained of the valve using the same threshold across all samples.

### Antibodies.

The following antibodies were used for immunostaining: KLF4 (1:100; R&D, AF3158), ERG (1:100; Abcam, ab92513), CD31/PECAM-1 (1:200; R&D, AF3628), GFP (1:100; Abcam, 6673), RFP (1:50; Rockland, p/n600-401-379), Ki67 (1:100; Abcam, ab16667), CD45 (1:150; R&D, AF114), p-SMAD2 (1:100; Millipore, AB3849-I), versican (1:150; Millipore, AB1033), and HIF1α (1:100; Novus, NB100-134).

### Statistics.

All studies were performed with a minimum of 3 biological replicates. Single-cell RNA-Seq included pooled tissue from 5 mice (mixed sexes) for each group and time point and was performed in duplicate. Data are presented as mean ± SD and were produced using GraphPad Prism version 9. Individual data points on graphs for valve leaflet area and immunostaining quantifications represent an individual mouse. *P* values were calculated using a 2-tailed unpaired *t* test or ANOVA with Tukey’s multiple-comparison test, as indicated in each figure legend. *P* values less than 0.05 were considered statistically significant.

### Study approval.

All experiments were performed in accordance with the guidelines of the Committee for Animal Research and were approved by the Institutional Animal Care and Use Committee at the University of Pennsylvania.

### Data availability.

Raw FASTQ files and processed data from scRNA-Seq were deposited to the Gene Expression Omnibus and can be accessed under accession number GSE254261. Values from graphed data are included in the Supporting Data Values file.

## Author contributions

JAP, LMG, CCH, WWH, and MLK conceptualized the study. JAP, LMG, CCH, LW, MC, JY, WY, KT, CT, and TK developed methodology. JAP, LMG, CCH, LW, YX, SG, MD, XC, CT, TK, MC, JY, and MLK performed investigation. JAP and MLK acquired funding. MLK and PM performed project administration. MLK supervised the study. JAP and MLK wrote the original draft. JAP, LMG, GF, KT, WWH, and MLK reviewed and edited the manuscript.

## Supplementary Material

Supplemental data

Supporting data values

## Version 1 06/16/2025

Electronic publication
