# Supplementary material for: Hemodynamic forces prevent myxomatous valve disease in mice through KLF2/4 signaling
Source: J Clin Invest. 2025 Jun 16;135(12):e186593. doi: 10.1172/JCI186593 (PMC12165810; doi:10.1172/JCI186593)
Supplement: Supplemental data [file jci-135-186593-s099.pdf]

Supplemental Material

Hemodynamic forces prevent myxomatous valve disease in mice through KLF2/4 signaling

Jesse Pace[1†], Lauren Goddard[1†], Courtney Hong[1], Liqing Wang[2], Jisheng Yang[1], Mei Chen[1], Yitian Xu[1], Martin Dominguez[1], Siqi Gao[1], Xiaowen Chen[1], Patricia Mericko[1], Can Tan[3], Tsutomu Kume[3], Wenbao Yu[4], Kai Tan[4], Wayne W. Hancock[2], Giovanni Ferrari[5], Mark L. Kahn[1*]

[1]Cardiovascular Institute, Department of Medicine, Perelman School of Medicine, University of Pennsylvania, Philadelphia, PA, USA.

[2]Division of Transplant Immunology, Department of Pathology and Laboratory Medicine, and Biesecker Center for Pediatric Liver Disease, Children's Hospital of Philadelphia and University of Pennsylvania, Philadelphia, PA, USA.

[3]Department of Medicine, Feinberg Cardiovascular and Renal Research Institute, Feinberg School of Medicine, Northwestern University, Chicago, IL, USA.

[4]Division of Oncology and Center for Childhood Cancer Research, Children's Hospital of Philadelphia, Philadelphia, PA, USA; Center for Single Cell Biology, Children's Hospital of Philadelphia, Philadelphia, PA, USA; Department of Pediatrics, Perelman School of Medicine, University of Pennsylvania, Philadelphia, PA, USA.

[5]Department of Surgery, Columbia University, New York, NY; Department of Biomedical Engineering, Columbia University, New York, NY, USA.

[†]These authors contributed equally to this work

*Corresponding author

Supplemental Methods

Bone Marrow Transplantation

For bone marrow transplantation studies, bone marrows were harvested from adult *Ccr2*$^{GFP/GFP}$ or *Ccr2*$^{GFP/+}$ mice from femur, tibia and humerus *(1)*. At 6 weeks of age, *Prox1*$^{CreERT2+}$*;Klf2*$^{fl/fl}$*;Klf4*$^{fl/fl}$ and *Klf2*$^{fl/fl}$*;Klf4*$^{fl/fl}$ mice were subjected to two doses of irradiation 4 hours apart, totaling 11.5 Gy. Irradiated recipient mice received $5x10^6$ cells per mouse via tail vein injection. Mice were treated with enrofloxacin for 2 weeks immediately following bone marrow transfer and induced with tamoxifen at 4 weeks post bone marrow transplant.

Single Cell Data Processing

Libraries were sequenced using an Illumina NextSeq500. Raw sequencing data were demultiplexed and aligned to a custom reference mouse genome containing an added reference for the *Cre* transcript using CellRanger on the 10X Genomics Cloud. Downstream analysis was performed using the R-package Seurat *(2)*. For quality control and filtering, only genes expressed in a minimum of 3 cells were retained. Cells expressing between 200 and 5000 genes were retained and cells that contained more than 15% mitochondrial genes were excluded. Reciprocal PCA method was performed to integrate the samples from different timepoints and conditions. Cluster labeling was performed manually based on established marker genes. Transcription factor enrichment analysis was performed using the chip enrichment analysis (ChEA) database on Enrichr, based on the top 50 differentially expressed genes upregulated in the valve endothelial cell cluster following loss of KLF2/4 *(3)*. The AddModuleScore function of Seurat was used to calculate a ‘SMAD score’ based on expression TGFb/SMAD2/3 target genes *(4, 5)*. For investigation of cell-cell communication in scRNAseq data, the R-packages CellChat and NicheNet were used with default paramaters *(6, 7)*.

Whole-mount staining of mitral valves

Whole-mount staining of mitral valves were performed on unloaded HHT hearts and controls 4 days post-transplant *(8, 9)*. Briefly, hearts were perfused with ice-cold PBS and then 4% PFA before being fixed overnight in 2% PFA at 4C. The dissected mitral valves were permeabilized in 0.3% Triton X-100 in PBS for 1 hour at 4C, blocked in 5% normal donkey serum (plus 0.5% BSA, 0.3% Triton X-100, and 0.1% NaN3 in PBS) for 2 hours at 4C, and then incubated with CD31/PECAM1 (R&D, AF3628), CDH5/VE-Cadherin (BD, 555289) diluted in blocking buffer for 3 days at 4C. Fluorochrome-conjugated secondary antibodies were incubated for 2 days at 4C. Samples were washed, postfixed with 4% PFA, and cleared with FocusClear (CelExplorer Labs, FC-101) and flat-mounted on slides in mounting medium. Imaging was performed on Nikon A1 Confocal Laser Microscope with the NIS-Elements Viewer software.

RNA Extraction and quantitative PCR

RNA extraction from valve tissue was performed using the RNeasy Micro kit (Qiagen). cDNA was synthesized from 150 ng total RNA using the Superscript III Reverse Transcriptase (Invitrogen). Real-time PCR was performed with Power SYBR Green PCR Master Mix (Applied Biosciences). The following primers were used for Klf2 F: 5’CGCCTCGGGTTCATTTC3’ R: 5’AGCCTATCTTGCCGTCCTTT3’ and Klf4 F: 5’GTGCCCCGACTAACCGTTG3’ R: 5’GTCGTTGAACTCCTCGGTCT3’ *(10)*. The following primers were used for Hif1a F:5’ACAAGTCACCACAGGACAG3’ and Hif1a R: 5’AGGGAGAAAATCAAGTCG3’, as well as Vegfa F: 5’CAGGCTGCTCTAACGATGAA3’ and Vegfa R: 5’CAGGAATCCCAGAAACAACC3’

RNAscope

RNAscope was performed using the RNAscope Multiplex Fluorescent v2 assay (ACDBio). Formalin-fixed paraffin embedded sections were used for this assay according to manufacturer's instructions. Briefly, slides were baked and deparaffinized before treatment with hydrogen peroxide, target retrieval reagent (15 minutes), and protease plus (30 minutes). Probes targeting human or mouse *KLF2 (*408711, 510671, ACDBio) and human or mouse *KLF4* (457461, 426711, ACDBio) were hybridized at 40C for 2 hours and then amplified and developed according to manufacturer's instructions.

Supplemental Figures

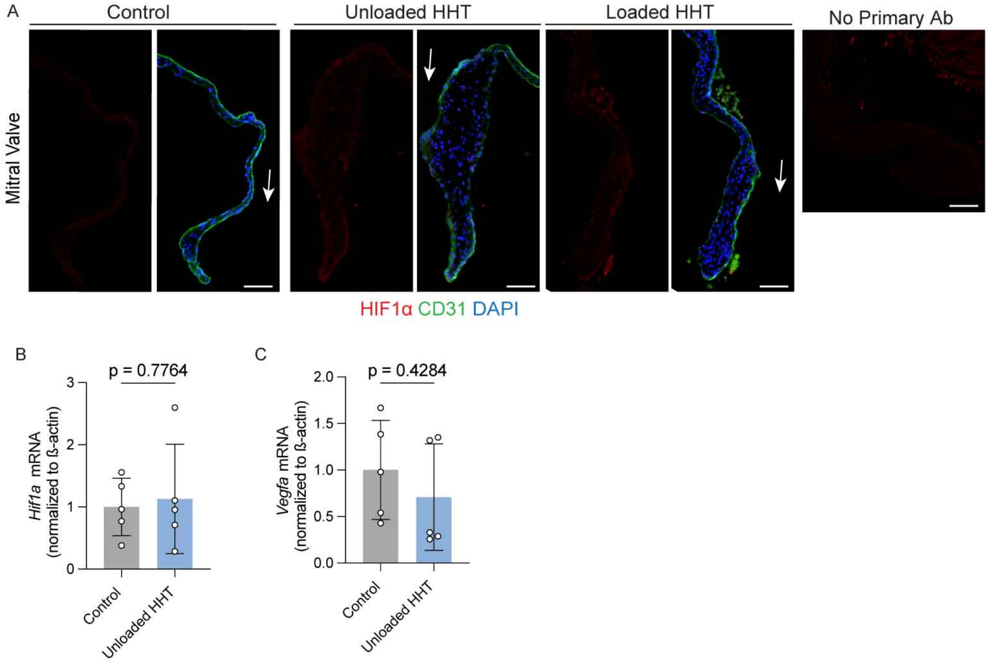

Figure S1: HIF1a and *Vegfa* are unchanged in mitral valves following HHT

(A) HIF1a and CD31 staining of mitral valve at 4 days after transplant. Arrows indicate the flow side of the valve. A no primary antibody control is shown. Scale bar = 50um.

(B) Quantitative RT-PCR for *Hif1a* on isolated mitral valves from unloaded HHT and control hearts 4 days after transplant. P-value shown on graph as calculated by unpaired t-test.

(C) Quantitative RT-PCR for *Vegfa* on isolated mitral valves from unloaded HHT and control hearts 4 days after transplant. P-value shown on graph as calculated by unpaired t-test.

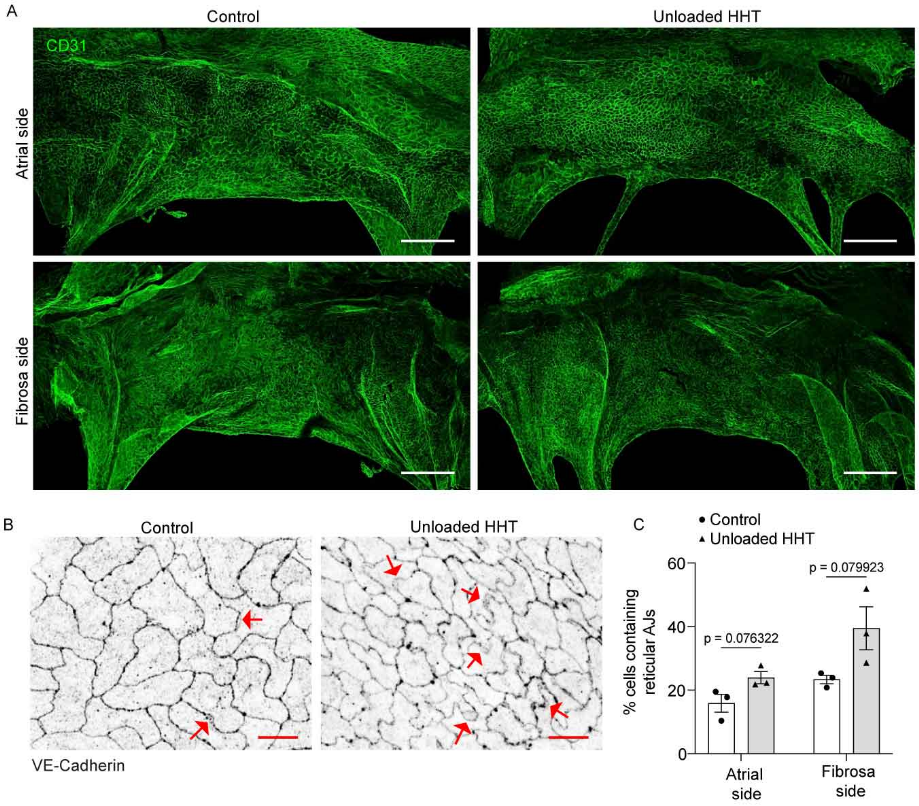

Figure S2: Valve endothelial cell junctions are altered following HHT

(A) CD31 whole mount immunostaining of mitral valve at 4 days after transplant. Images shown are from both the atrial and fibrosa aspects of the posterior mitral valve leaflet.

(B) VE-Cadherin immunostaining of mitral valve at 4 days after transplant.

(C) Quantification of adherens junctions at 4 days after transplant in B. P-values are shown as calculated by unpaired t-tests.

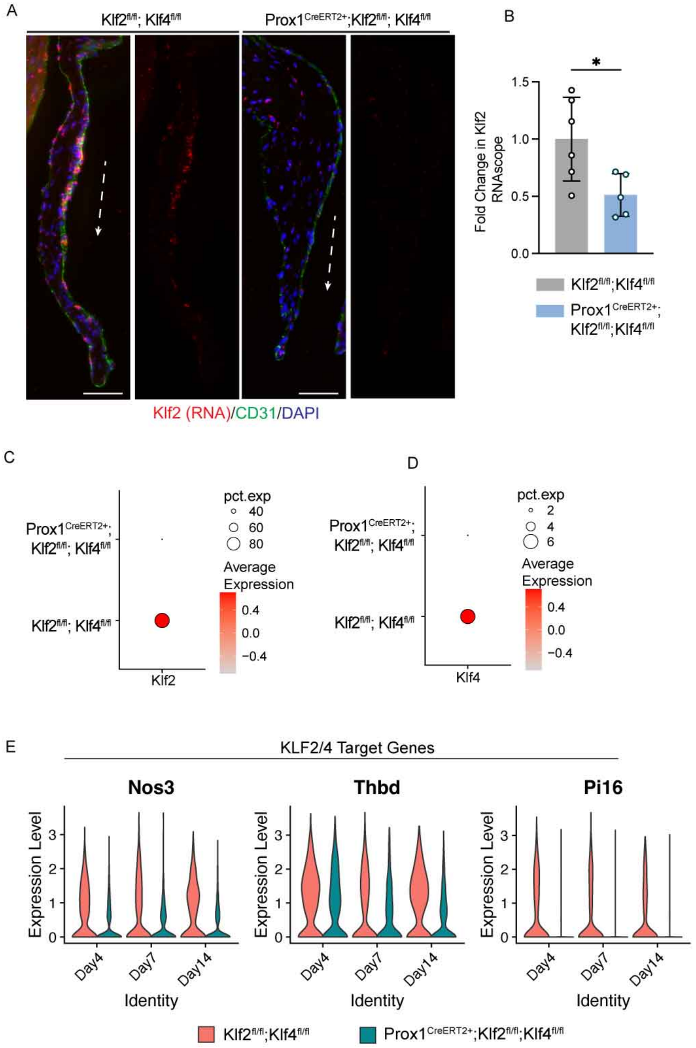

Figure S3: Validation of KLF2/4 deletion in *Prox1$^{CreERT2+}$;Klf2$^{fl/fl}$;Klf4$^{fl/fl}$* mice

(A) In-situ hybridization for *Klf2* mRNA costained with CD31 and DAPI in mitral valves of *Klf2$^{fl/fl}$;Klf4$^{fl/fl}$* and *Prox1$^{CreERT2+}$;Klf2$^{fl/fl}$;Klf4$^{fl/fl}$* mice at 4 days post tamoxifen treatment. Scale bar = 50um.

(B) Quantification of *Klf2* percent area stained in (A). *p<0.05, as calculated by unpaired t-test.

(C) DotPlot from single cell RNA-sequencing data showing levels of *Klf2* in valve endothelial cell populations of *Prox1$^{CreERT2+}$;Klf2$^{fl/fl}$;Klf4$^{fl/fl}$* and *Klf2$^{fl/fl}$;Klf4$^{fl/fl}$* mice.

(D) DotPlot from single cell RNA-sequencing data showing levels of *Klf4* in valve endothelial cell populations of *Prox1$^{CreERT2+}$;Klf2$^{fl/fl}$;Klf4$^{fl/fl}$* and *Klf2$^{fl/fl}$;Klf4$^{fl/fl}$* mice.

(E) Violin plots of KLF2/4 target genes Nos3, Thbd, and Pi16, which are significantly decreased following deletion of Klf2/4.

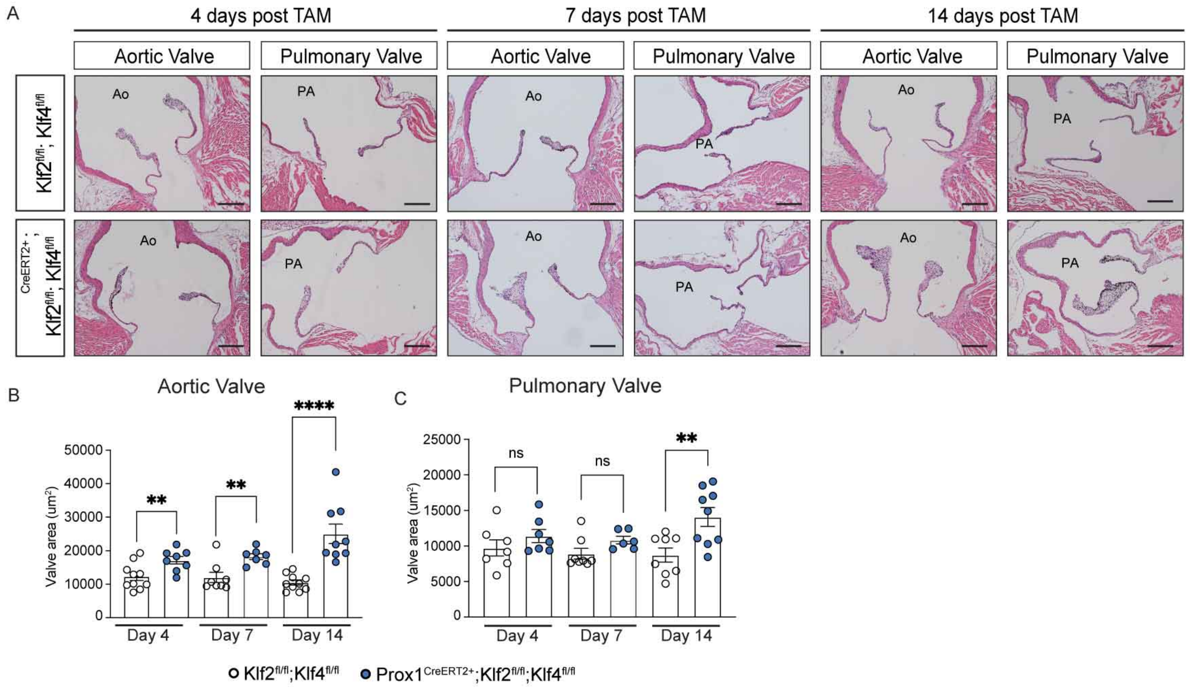

Figure S4: Myxomatous phenotypes in $\mathit{Prox1}^{CreERT2+}$*;Klf2*$^{fl/fl}$*;Klf4*$^{fl/fl}$ aortic and pulmonary valves

(A) H-E staining of heart tissue from $\mathit{Prox1}^{CreERT2+}$*;Klf2*$^{fl/fl}$*;Klf4*$^{fl/fl}$ and *Klf2*$^{fl/fl}$*;Klf4*$^{fl/fl}$ mice at days 4, 7 and 14 post tamoxifen treatment. Scale bar = 100um.

(B) Quantification of valve leaflet area at days 4, 7 and 14 post tamoxifen treatment. N=6 mice per group.**p<0.01, **** p<0.0001, as calculated by unpaired t-tests. Ao, aortic outflow tract, PA, pulmonary artery

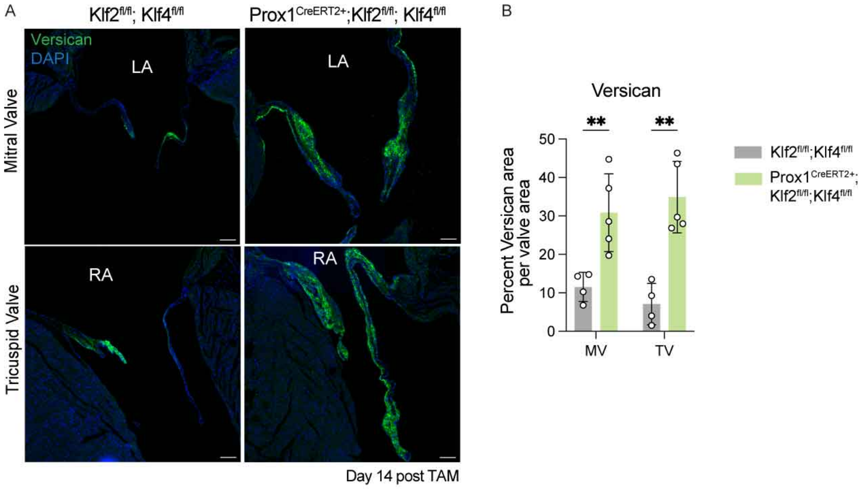

Figure S5: Versican abundance is increased in *Prox1$^{CreERT2+}$;Klf2$^{fl/fl}$;Klf4$^{fl/fl}$* mice

(A) Immunostaining for Versican in tricuspid and mitral valves from *Prox1$^{CreERT2+}$;Klf2$^{fl/fl}$* and *Prox1$^{CreERT2+}$;Klf2$^{fl/fl}$;Klf4$^{fl/+}$* mice at 14 days post tamoxifen. Scale bar = 50um.

(B) Quantification of valve Versican staining in (A). **p<0.01, as calculated by unpaired t-tests.

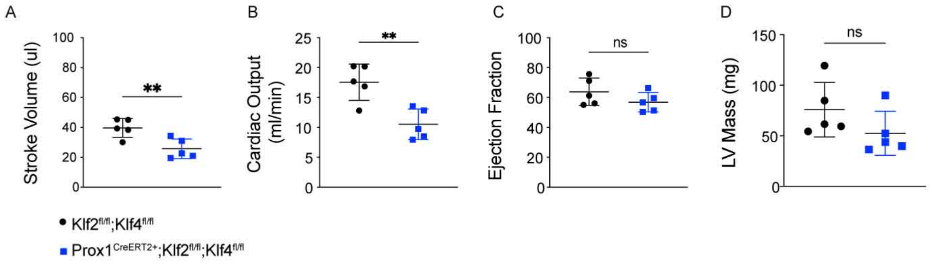

E

	Valve regurgitation or stenosis				Effusion		
	AV	PV	MV	TV	Pericardial	Pleural	Ascites
$Klf2^{fl/fl}$;$Klf4^{fl/fl}$	N	N	N	N	N	N	N
$Klf2^{fl/fl}$;$Klf4^{fl/fl}$	N	N	N	N	N	N	N
$Klf2^{fl/fl}$;$Klf4^{fl/fl}$	N	N	N	N	N	N	N
$Prox1^{CreERT2+}$;$Klf2^{fl/fl}$;$Klf4^{fl/fl}$	N	PR (mild)	N	TR(mild)	Y	Y	Y
$Prox1^{CreERT2+}$;$Klf2^{fl/fl}$;$Klf4^{fl/fl}$	N	N	N	N	Y	Y	Y
$Prox1^{CreERT2+}$;$Klf2^{fl/fl}$;$Klf4^{fl/fl}$	N	N	N	N	Y	Y	Y
$Prox1^{CreERT2+}$;$Klf2^{fl/fl}$;$Klf4^{fl/fl}$	N	N	N	N	N	N	N

Figure S6: Echocardiography of *$Prox1^{CreERT2+}$;$Klf2^{fl/fl}$;$Klf4^{fl/fl}$* mice

(A) Stroke volume of *$Klf2^{fl/fl}$;$Klf4^{fl/+}$* mice and *$Prox1^{CreERT2+}$;$Klf2^{fl/fl}$;$Klf4^{fl/+}$* mice 14 days post tamoxifen. **p<0.01, as calculated by unpaired t-test.

(B) Cardiac output in *$Klf2^{fl/fl}$;$Klf4^{fl/+}$* mice and *$Prox1^{CreERT2+}$;$Klf2^{fl/fl}$;$Klf4^{fl/+}$* mice 14 days post tamoxifen. **p<0.01, as calculated by unpaired t-test.

(C) Cardiac ejection fraction in *$Klf2^{fl/fl}$;$Klf4^{fl/+}$* mice and *$Prox1^{CreERT2+}$;$Klf2^{fl/fl}$;$Klf4^{fl/+}$* mice 14 days post tamoxifen.

(D) Left ventricular mass in *$Klf2^{fl/fl}$;$Klf4^{fl/+}$* mice and *$Prox1^{CreERT2+}$;$Klf2^{fl/fl}$;$Klf4^{fl/+}$* mice 14 days post tamoxifen.

(E) Echocardiographic findings of *$Klf2^{fl/fl}$;$Klf4^{fl/+}$* mice and *$Prox1^{CreERT2+}$;$Klf2^{fl/fl}$;$Klf4^{fl/+}$* mice 14 days post tamoxifen.

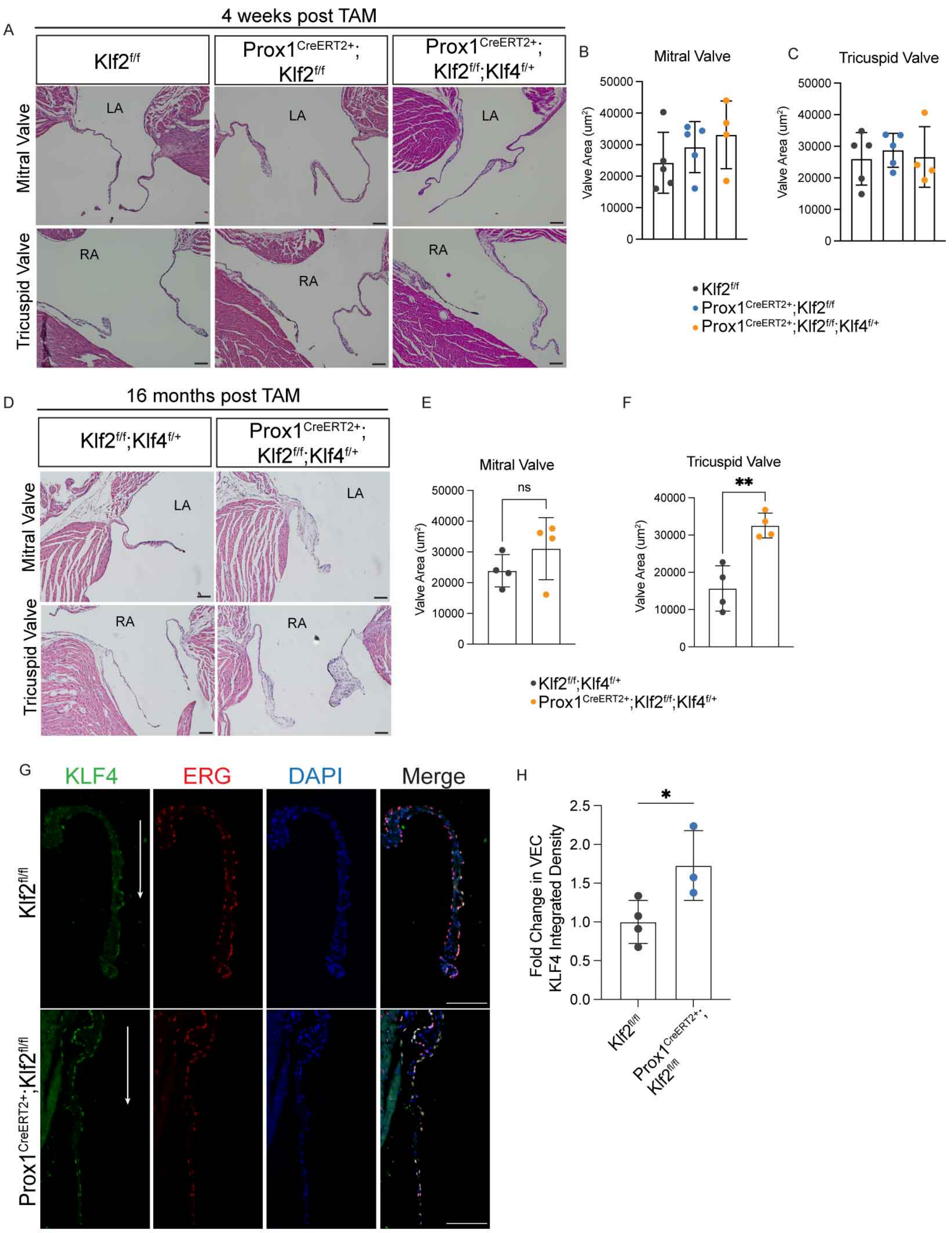

Figure S7: Deletion of *Klf2* or *Klf2* and one allele of *Klf4* does not confer a valve phenotype.

(A) H-E staining of tricuspid and mitral valves from *Prox1*$^{CreERT2+}$*;Klf2*$^{fl/fl}$ and *Prox1*$^{CreERT2+}$*;Klf2*$^{fl/fl}$*;Klf4*$^{fl/+}$ mice at 4 weeks post tamoxifen.

(B) Quantification of mitral valve size in the indicated animals. Each dot represents one mouse.

(C) Quantification of tricuspid valve size in the indicated animals. Each dot represents one mouse.
(D) H-E staining of tricuspid and mitral valve from *Prox1*$^{CreERT2+}$;*Klf2*$^{fl/fl}$;*Klf4*$^{fl/+}$ mice at 16 months post tamoxifen.
(E) Quantification of mitral valve size in the indicated animals. ns, not significant. N=4 mice per group. P>0.05, as calculated by unpaired t-test.
(F) Quantification of tricuspid valve size in the indicated animals. N=4 mice per group.**P<0.01, as calculated by unpaired t-test.
(G) Immunostaining for KLF4, ERG, and DAPI in from *Klf2*$^{fl/fl}$ and *Prox1*$^{CreERT2+}$;*Klf2*$^{fl/fl}$ mice 4 weeks post tamoxifen. Arrow indicates flow side of the valve. Scale bar = 50um.
(H) Quantification of valve endothelial KLF4 in (G). *p<0.05, as calculated by unpaired t-test.

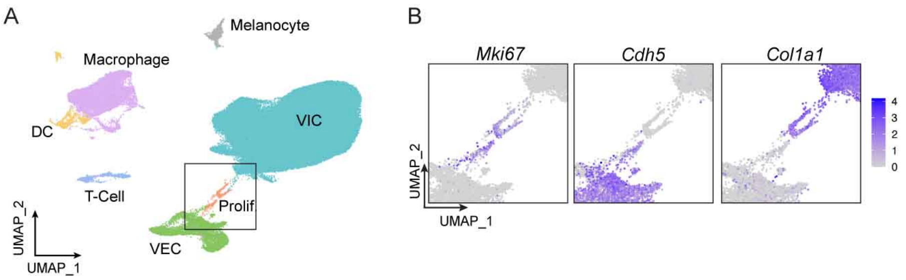

Figure S8: Proliferating cell cluster includes distinct populations of proliferating VEC and VIC

(A) UMAP showing the annotated clusters from all timepoints and groups of *Prox1*$^{CreERT2+}$*;Klf2*$^{fl/fl}$*;Klf4*$^{fl/fl}$ and *Klf2*$^{fl/fl}$*;Klf4*$^{fl/fl}$ mice.

(B) Feature Plots showing expression of *Mki67*, *Cdh5* and *Col1a1* within the proliferating cell cluster.

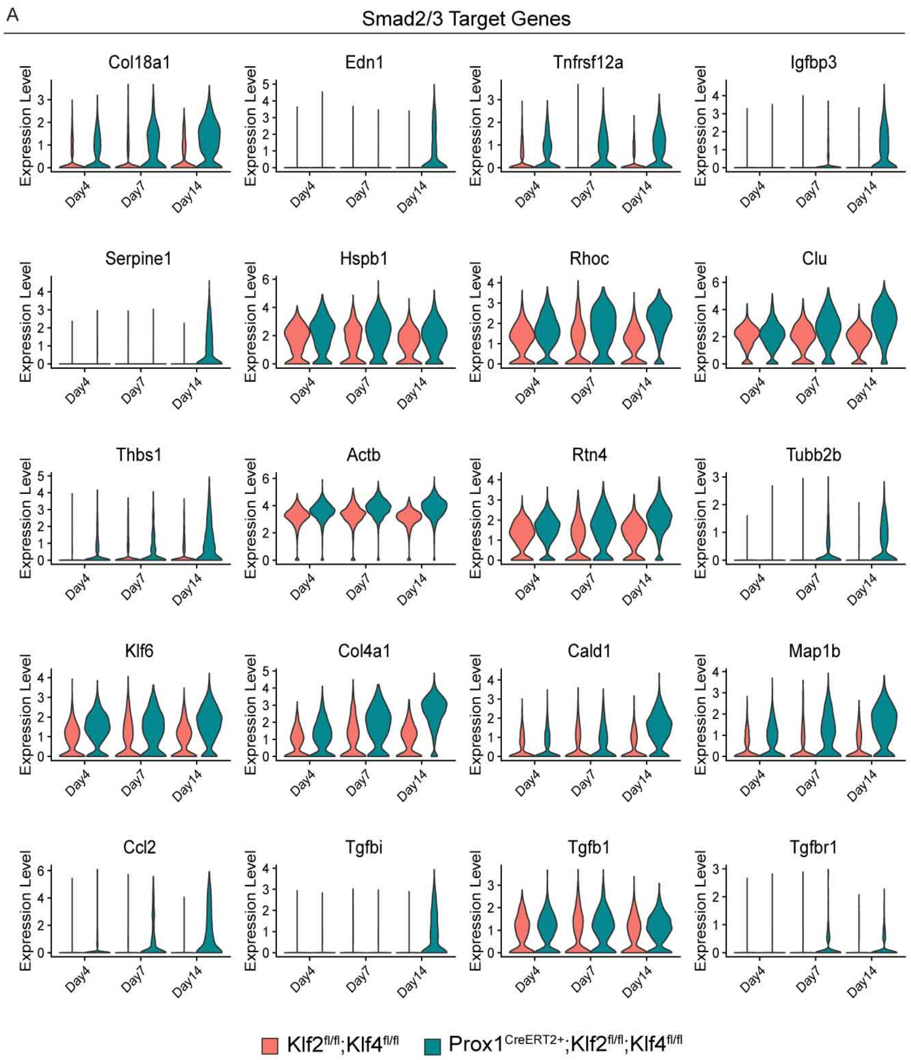

Figure S9: Smad2/3 target genes upregulated in *Prox1$^{CreERT2+}$;Klf2$^{fl/fl}$;Klf4$^{fl/fl}$* mice

(A) Violin plots showing the expression of genes upregulated in *Prox1$^{CreERT2+}$;Klf2$^{fl/fl}$;Klf4$^{fl/fl}$* mice that are Smad2/3 target genes.

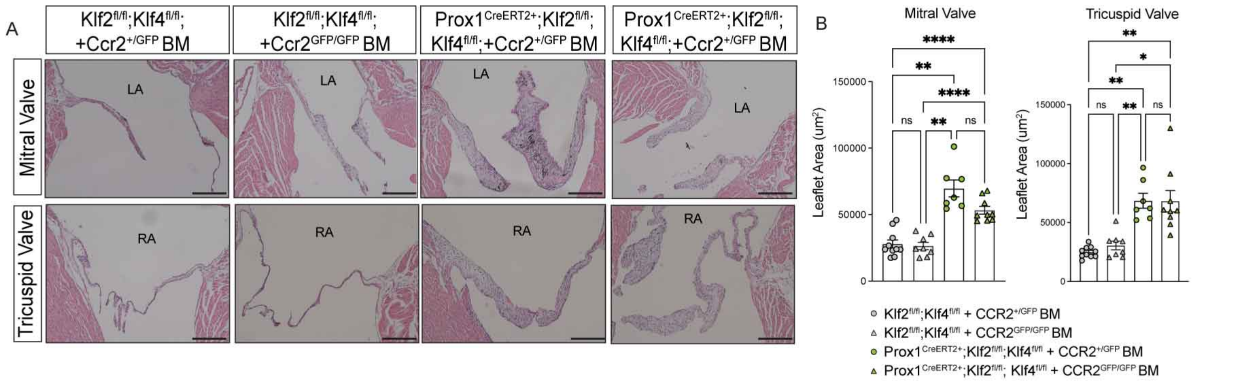

Figure S10: Transplantation of CCR2-deficient bone marrow does not prevent myxomatous valve formation in *Prox1$^{CreERT2+}$;Klf2$^{fl/fl}$;Klf4$^{fl/fl}$* mice

(A) H&E staining of mitral and tricuspid valve at day 14 post tamoxifen. Scale bar = 100um.

(B) Quantification of valve leaflet area from histology in the indicated animals. N=7 mice per group minimum. *p<0.05, **p<0.01, ****p<0.0001, as calculated by two-way ANOVA with Tukey's multiple comparison tests.

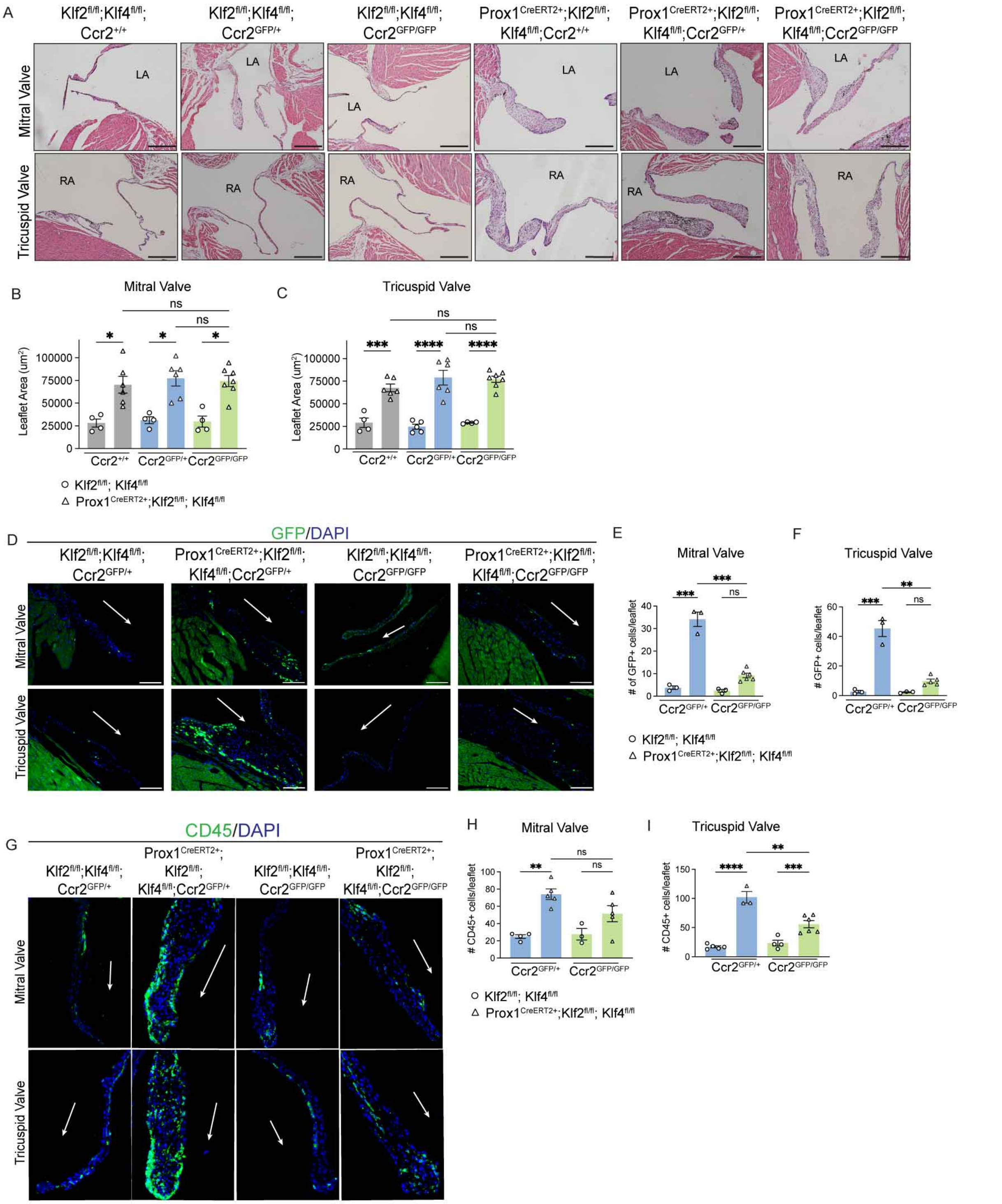

Figure S11: Genetic loss of CCR2 does not prevent myxomatous valve formation in *Prox1$^{CreERT2+}$;Klf2$^{fl/fl}$;Klf4$^{fl/fl}$* mice

(A) H-E staining of mitral and tricuspid valve at day 14 post tamoxifen. Scale bar = 100um.

(B) Quantification of mitral valve leaflet area from histology in the indicated animals. N=4 mice per group minimum. *$p<0.05$, as calculated by two-way ANOVA with Tukey's multiple comparison tests.

(C) Quantification of tricuspid valve leaflet area from histology in the indicated animals. N=4 mice per group minimum. ***p<0.001, ****p<0.0001, as calculated by two-way ANOVA with Tukey's multiple comparison tests.

(D) Immunostaining for GFP in Prox1$^{CreERT2+}$;Klf2$^{fl/fl}$;Klf4$^{fl/fl}$ and Klf2$^{fl/fl}$;Klf4$^{fl/fl}$ mice with partial or complete loss of CCR2 at day 14 post tamoxifen. Scale bar = 100um.

(E) Quantification of percentage of GFP+ cells in mitral valve leaflets at day 14 post tamoxifen. N=3 mice per group minimum. *p<0.05, **p<0.01,***p<0.001, ****p<0.0001, as calculated by two-way ANOVA with Tukey's multiple comparison tests.

(F) Quantification of percentage of GFP+ cells in tricuspid valve leaflets at day 14 post tamoxifen. *p<0.05, **p<0.01,***p<0.001, ****p<0.0001, as calculated by two-way ANOVA with Tukey's multiple comparison tests.

(G) Immunostaining for CD45 in Prox1$^{CreERT2+}$;Klf2$^{fl/fl}$;Klf4$^{fl/fl}$ and Klf2$^{fl/fl}$;Klf4$^{fl/fl}$ mice with partial or complete loss of CCR2 at day 14 post tamoxifen. Scale bar = 100um.

(H) Quantification of percentage of CD45+ cells in mitral valve leaflets at day 14 post tamoxifen. N=3 mice per group minimum. *p<0.05, **p<0.01,***p<0.001, ****p<0.0001, as calculated by two-way ANOVA with Tukey's multiple comparison tests.

(I) Quantification of percentage of CD45+ cells in tricuspid valve leaflets at day 14 post tamoxifen. N=3 mice per group minimum. *p<0.05, **p<0.01,***p<0.001, ****p<0.0001, as calculated by two-way ANOVA with Tukey's multiple comparison tests.

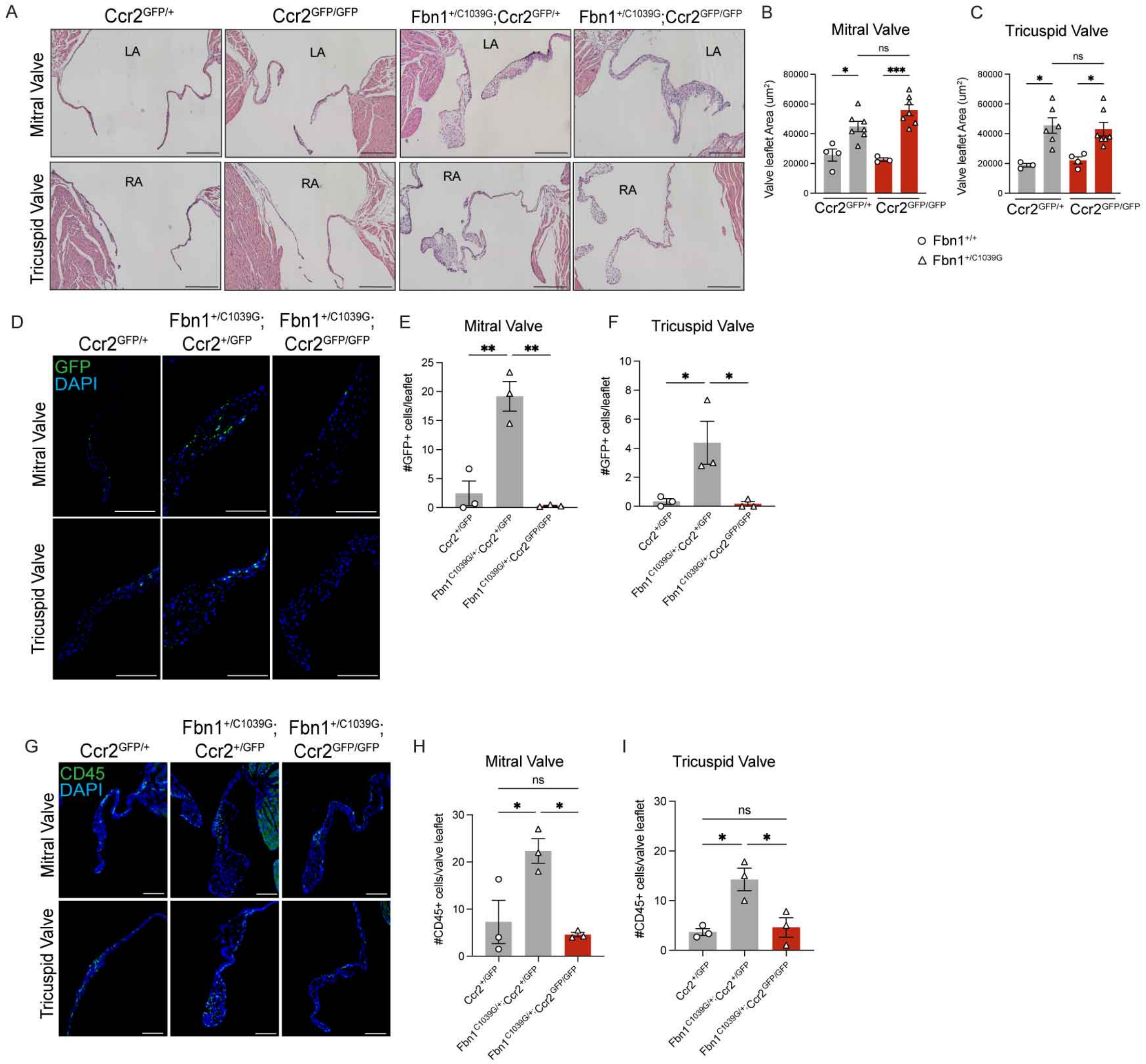

Figure S12: Genetic loss of CCR2 does not rescue myxomatous valve formation in *Fbn1*

(A) H&E staining of tricuspid and mitral valves of *$Fbn1^{+/C1039G}$;$Ccr2^{GFP/GFP}$* and *$Fbn1^{+/C1039G}$;$Ccr2^{GFP/+}$* mice at 2 months of age. Scale bar = 100um.

(B) Quantification of mitral valve leaflet area from staining in the indicated animals. N=3 mice per group minimum. *p<0.05, **p<0.01, ***p<0.001, as calculated by two-way ANOVA with Tukey's multiple comparison tests.

(C) Quantification of tricuspid valve leaflet area from staining in the indicated animals. N=3 mice per group minimum. *p<0.05, **p<0.01, ***p<0.001, as calculated by two-way ANOVA with Tukey's multiple comparison tests.

(D) Immunostaining for GFP in F*$bn1^{+/C1039G}$* mice. Scale bar = 100um.

(E) Quantification of percentage of GFP+ cells in mitral valve leaflets at 2 months of age. N=3 mice per group minimum. *p<0.05, **p<0.01, as calculated by one-way ANOVA with Tukey's multiple comparison tests.

(F) Quantification of percentage of GFP+ cells in tricuspid valve leaflets at 2 months of age. N=3 mice per group minimum. *p<0.05, **p<0.01, as calculated by one -way ANOVA with Tukey's multiple comparison tests.

(G) Immunostaining for CD45 in F*bn1*$^{+/C1039G}$ mice. Scale bar = 100um.

(H) Quantification of percentage of CD45+ cells in mitral valve leaflets at 2 months of age. N=3 mice per group minimum. *p<0.05, **p<0.01, as calculated by one -way ANOVA with Tukey's multiple comparison tests.

(I) Quantification of percentage of CD45+ cells in tricuspid valve leaflets at 2 months of age. N=3 mice per group minimum. *p<0.05, **p<0.01, as calculated by one -way ANOVA with Tukey's multiple comparison tests. LA, left atrium, RA, right atrium

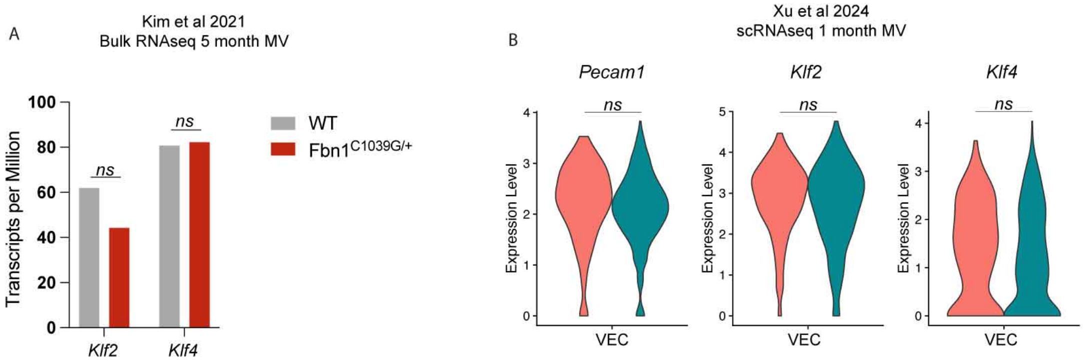

Figure S13: *Klf2/4* expression is unchanged in *Fbn1*$^{C1039G/+}$ mice

(A) Analysis of *Klf2/4* expression from bulk RNA-sequencing of 5-month-old mitral valve from Fbn1$^{C1039G/+}$ mice and controls from Kim et al 2021 (data retrieved from GSE137681).

(B) Analysis of VEC *Klf2/4* expression from scRNA-sequencing of 1-month-old mitral valve from Fbn1$^{C1039G/+}$ mice and controls from Xu et al 2021 (data retrieved from GSE261874).

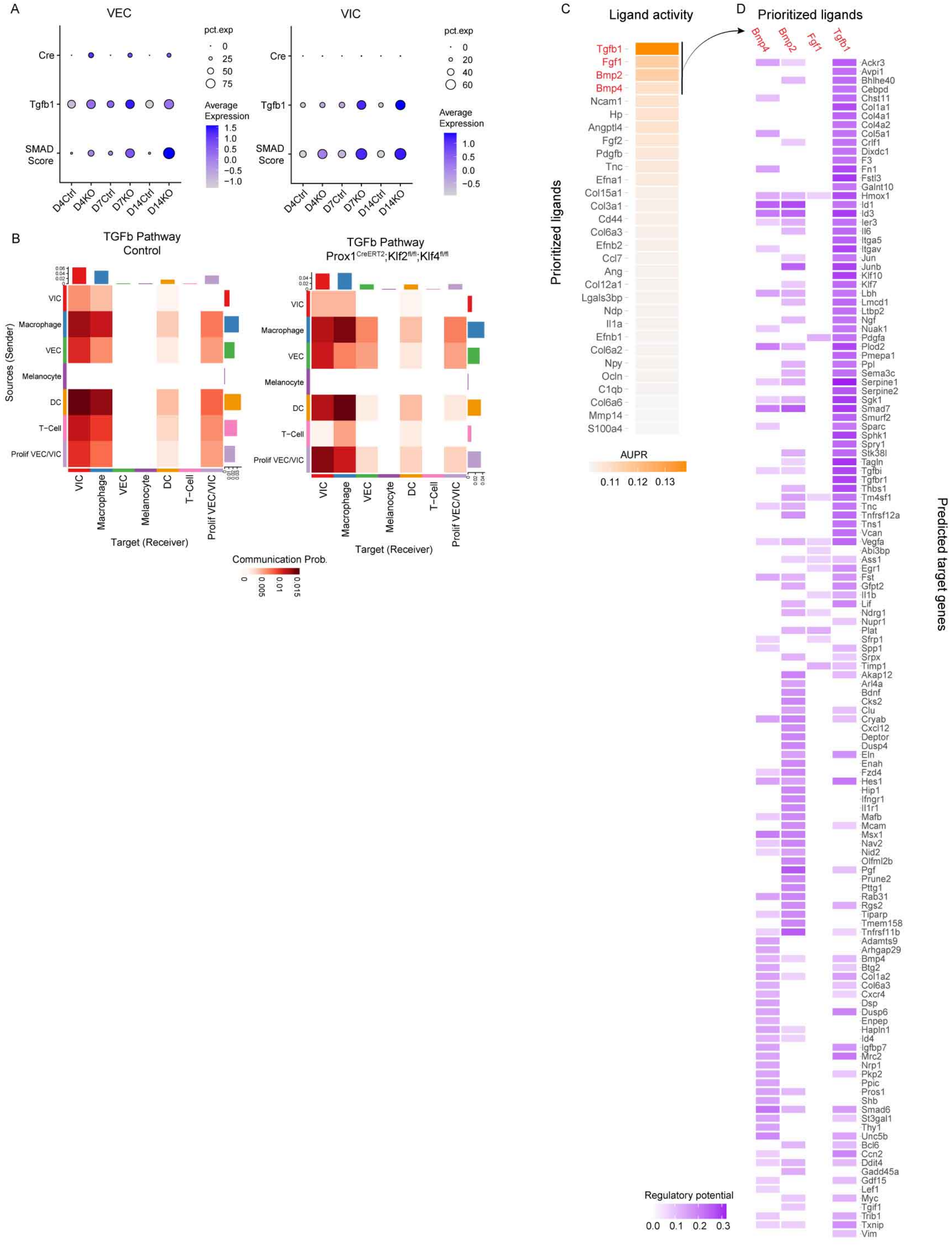
A
VEC
VIC
Cre
Tgfb1
SMAD Score
pct.exp
Average Expression
D4Ctrl
D4KO
D7Ctrl
D7KO
D14Ctrl
D14KO
B
TGFb Pathway Control
TGFb Pathway Prox1CreERT2;Klf2fl/fl;Klf4fl/fl
Sources (Sender)
Target (Receiver)
VIC
Macrophage
VEC
Melanocyte
DC
T-Cell
Prolif VEC/VIC
Communication Prob.
C
Ligand activity
Prioritized ligands
Tgfb1
Fgf1
Bmp2
Bmp4
Ncam1
Hp
Angptl4
Fgf2
Pdgfb
Tnc
Efna1
Col15a1
Col3a1
Cd44
Col6a3
Efnb2
Ccl7
Ang
Col12a1
Lgals3bp
Ndp
Il1a
Efnb1
Col6a2
Npy
Ocln
C1qb
Col6a6
Mmp14
S100a4
AUPR
0.11 0.12 0.13
D
Prioritized ligands
Bmp4
Bmp2
Fgf1
Tgfb1
Predicted target genes
Ackr3
Avpi1
Bhlhe40
Cebpd
Chst11
Col1a1
Col4a1
Col4a2
Col5a1
Crlf1
Dixdc1
F3
Fn1
Fstl3
Galnt10
Hmox1
Id1
Id3
Ier3
Il6
Itga5
Itgav
Jun
Junb
Klf10
Klf7
Lbh
Lmcd1
Ltbp2
Ngf
Nuak1
Pdgfa
Plod2
Pmepa1
Ppl
Sema3c
Serpine1
Serpine2
Sgk1
Smad7
Smurf2
Sparc
Sphk1
Spry1
Stk38l
Tagln
Tgfbi
Tgfbr1
Thbs1
Tm4sf1
Tnc
Tnfrsf12a
Tns1
Vcan
Vegfa
Abi3bp
Ass1
Egr1
Fst
Gfpt2
Il1b
Lif
Ndrg1
Nupr1
Plat
Sfrp1
Spp1
Srpx
Timp1
Akap12
Arl4a
Bdnf
Cks2
Clu
Cryab
Cxcl12
Deptor
Dusp4
Eln
Enah
Fzd4
Hes1
Hip1
Ifngr1
Il1r1
Mafb
Mcam
Msx1
Nav2
Nid2
Olfml2b
Pgf
Prune2
Pttg1
Rab31
Rgs2
Tiparp
Tmem158
Tnfrsf11b
Adamts9
Arhgap29
Bmp4
Btg2
Col1a2
Col6a3
Cxcr4
Dsp
Dusp6
Enpep
Hapln1
Id4
Igfbp7
Mrc2
Nrp1
Pkp2
Ppic
Pros1
Shb
Smad6
St3gal1
Thy1
Unc5b
Bcl6
Ccn2
Ddit4
Gadd45a
Gdf15
Lef1
Myc
Tgif1
Trib1
Txnip
Vim
Regulatory potential
0.0 0.1 0.2 0.3

Figure S14: Cell-cell communication analysis of scRNAseq data from *Prox1$^{CreERT2+}$;Klf2$^{fl/fl}$;Klf4$^{fl/fl}$* and *Klf2$^{fl/fl}$;Klf4$^{fl/fl}$* mice

(A) DotPlot of valve endothelial and valve interstitial cells from scRNAseq data showing expression of Cre recombinase, Tgfb1, and calculated SMAD score based on expression of established TGFb/SMAD2/3 target genes. Values are split by genotype (Ctrl: *Klf2$^{fl/fl}$;Klf4$^{fl/fl}$* and KO: *Prox1$^{CreERT2+}$;Klf2$^{fl/fl}$;Klf4$^{fl/fl}$)* and timepoint post tamoxifen (D4: day 4 post tamoxifen, D7: day 7 post tamoxifen, and D14: day 14 post tamoxifen).

(B) Heatmap showing TGFb pathway signaling enrichment between different source and target cell types as calculated using CellChat.

(C) Heatmap of top ranked ligands signaling to valve interstitial cells in *Prox1$^{CreERT2+}$;Klf2$^{fl/fl}$;Klf4$^{fl/fl}$* mice compared to *Klf2$^{fl/fl}$;Klf4$^{fl/fl}$* mice determined through NicheNet. Ligands are prioritized as calculated by the area under the precision-recall curve (AUPR) between a ligand's target predictions and the observed transcriptional response.

(D) Top 4 prioritized ligands from (C) and predicted target genes based on NicheNet analysis are shown. Color scale indicates regulatory potential.

References

1. E. Park, M. A. Evans, H. Doviak, K. Horitani, H. Ogawa, Y. Yura, Y. Wang, S. Sano, K. Walsh, Bone Marrow Transplantation Procedures in Mice to Study Clonal Hematopoiesis. *J Vis Exp* , 10.3791/61875 (2021).

2. Y. Hao, S. Hao, E. Andersen-Nissen, W. M. Mauck, S. Zheng, A. Butler, M. J. Lee, A. J. Wilk, C. Darby, M. Zager, P. Hoffman, M. Stoeckius, E. Papalexi, E. P. Mimitou, J. Jain, A. Srivastava, T. Stuart, L. M. Fleming, B. Yeung, A. J. Rogers, J. M. McElrath, C. A. Blish, R. Gottardo, P. Smibert, R. Satija, Integrated analysis of multimodal single-cell data. *Cell* **184**, 3573-3587.e29 (2021).

3. E. Y. Chen, C. M. Tan, Y. Kou, Q. Duan, Z. Wang, G. V. Meirelles, N. R. Clark, A. Ma'ayan, Enrichr: interactive and collaborative HTML5 gene list enrichment analysis tool. *BMC Bioinformatics* **14**, 128 (2013).

4. R. Satija, J. A. Farrell, D. Gennert, A. F. Schier, A. Regev, Spatial reconstruction of single-cell gene expression data. *Nat Biotechnol* **33**, 495–502 (2015).

5. A. Butler, P. Hoffman, P. Smibert, E. Papalexi, R. Satija, Integrating single-cell transcriptomic data across different conditions, technologies, and species. *Nat Biotechnol* **36**, 411–420 (2018).

6. Inference and analysis of cell-cell communication using CellChat | Nature Communications (available at https://www.nature.com/articles/s41467-021-21246-9).

7. R. Browaeys, W. Saelens, Y. Saeys, NicheNet: modeling intercellular communication by linking ligands to target genes. *Nat Methods* **17**, 159–162 (2020).

8. C. Tan, Z.-D. Ge, S. Kurup, Y. Dyakiv, T. Liu, W. A. Muller, T. Kume, FOXC1 and FOXC2 Ablation Causes Abnormal Valvular Endothelial Cell Junctions and Lymphatic Vessel Formation in Myxomatous Mitral Valve Degeneration. *Arterioscler Thromb Vasc Biol* **44**, 1944–1959 (2024).

9. C. Tan, P. R. Norden, W. Yu, T. Liu, N. Ujiie, S. K. Lee, X. Yan, Y. Dyakiv, K. Aoto, S. Ortega, I. G. De Plaen, V. Sampath, T. Kume, Endothelial FOXC1 and FOXC2 promote intestinal regeneration after ischemia-reperfusion injury. *EMBO Rep* **24**, e56030 (2023).

10. L. M. Goddard, A.-L. Duchemin, H. Ramalingan, B. Wu, M. Chen, S. Bamezai, J. Yang, L. Li, M. P. Morley, T. Wang, M. Scherrer-Crosbie, D. B. Frank, K. A. Engleka, S. C. Jameson, E. E. Morrisey, T. J. Carroll, B. Zhou, J. Vermot, M. L. Kahn, Hemodynamic Forces Sculpt Developing Heart Valves through a KLF2-WNT9B Paracrine Signaling Axis. *Developmental Cell* **43**, 274-289.e5 (2017).
